# Transcriptome analysis and functional characterization of cerebral organoids in bipolar disorder

**DOI:** 10.1186/s13073-020-00733-6

**Published:** 2020-04-19

**Authors:** Annie Kathuria, Kara Lopez-Lengowski, Magdalena Vater, Donna McPhie, Bruce M. Cohen, Rakesh Karmacharya

**Affiliations:** 1 Center for Genomic Medicine, Massachusetts General Hospital, Harvard Medical School, Boston, MA USA; 2 Chemical Biology Program, Broad Institute of MIT & Harvard, Cambridge, MA USA; 3 Department of Psychiatry, Harvard Medical School, Boston, MA USA; 4 Schizophrenia and Bipolar Disorder Program, McLean Hospital, Belmont, MA USA; 5 Program in Neuroscience, Harvard University, Cambridge, MA USA; 6 Program in Chemical Biology, Harvard University, Cambridge, MA USA; 7 Harvard Stem Cell Institute, Cambridge, MA USA

**Keywords:** iPSC, Organoid, Bipolar disorder, Endoplasmic reticulum, MAM, NCAN, Cell adhesion, Immune signaling

## Abstract

**Background:**

Reprogramming human induced pluripotent stem cells (iPSCs) from somatic cells and generating three-dimensional brain organoids from these iPSCs provide access to live human neuronal tissue with disease-specific genetic backgrounds.

**Methods:**

Cerebral organoids were generated from iPSCs of eight bipolar disorder (BPI) patients and eight healthy control individuals. RNA-seq experiments were undertaken using RNA isolated from the cerebral organoids. Functional activity in the cerebral organoids was studied using microelectrode arrays.

**Results:**

RNA-seq data comparing gene expression profiles in the cerebral organoids showed downregulation of pathways involved in cell adhesion, neurodevelopment, and synaptic biology in bipolar disorder along with upregulation of genes involved in immune signaling. The central hub in the network analysis was neurocan (NCAN), which is located in a locus with evidence for genome-wide significant association in BPI. Gene ontology analyses suggested deficits related to endoplasmic reticulum biology in BPI, which was supported by cellular characterization of ER–mitochondria interactions. Functional studies with microelectrode arrays revealed specific deficits in response to stimulation and depolarization in BPI cerebral organoids.

**Conclusions:**

Our studies in cerebral organoids from bipolar disorder showed dysregulation in genes involved in cell adhesion, immune signaling, and endoplasmic reticulum biology; implicated a central role for the GWAS hit NCAN in the biology of BPI; and showed evidence of deficits in neurotransmission.

## Background

Investigations that focused on the biology of psychiatric disorders present significant scientific and methodological challenges. A primary barrier to scientific investigations of psychiatric disorders has been the infeasibility of accessing live neuronal tissue from patients to undertake experiments in the laboratory. Bipolar disorder (BPI) is a psychiatric disorder that is a significant contributor to the global burden of disease [1, 2] and is characterized by recurring episodes of depression and mania which affect perception, emotion, thought, and social behavior [3]. Its high prevalence, onset in young adulthood, and frequently chronic course with elevated morbidity and mortality rate make BPI a major mental health problem globally [4]. Genetic, twin, and clinical data show a strong genetic component to the etiology of BPI, with heritability estimates of more than 70% [5–7]. Despite its high prevalence and enormous impact, the treatment of BPI remains empirical, and a third of patients do not respond to multiple trials of different medications [8].

Due to the lack of causal genes with strong effect sizes, traditional genetic approaches cannot be employed effectively in studying the mechanistic underpinnings of the BPI disease biology. The discovery of a way to reprogram somatic cells to pluripotency using defined factors has opened new avenues for the study of disorders affecting the human brain [9]. iPSCs can be differentiated along the neuronal lineage to generate a range of neuronal cell types and tissue that have disease-specific genetic backgrounds [10, 11]. These approaches are now being applied to investigations of the disease biology of psychiatric disorders, including BPI [12–15]. Most studies to date have utilized two-dimensional neuronal cultures generated from iPSCs of BPI patients. New research on the self-organizing abilities of iPSC-derived progenitor cells has resulted in robust methods to generate three-dimensional brain organoids from human iPSCs [16, 17]. Cerebral organoids from human iPSCs recapitulate human cortical development and contain many neuronal and glial subtypes found in the human brain, including mature cortical neuron subtypes and synapses as well as networks of connectivity between different cells in the self-organizing structures [18–20].

Recent genome-wide association studies (GWAS) of BPI have shed some light on genetic loci with significant association for BPI. These loci include a number of genes that code for genes involved in neurodevelopment, synaptic biology, and neuronal excitability [21, 22]. In this study, we used iPSCs from patients with BPI and healthy control individuals to grow cerebral organoids ex vivo and investigate the disease biology of BPI. We generated cerebral organoids, grew them in culture for 6 months, and carried out immunohistochemical studies to identify the types of neuronal cells in the organoids. We undertook total RNA-seq experiments with the cerebral organoids to identify disease-specific transcriptomic differences. Analyses of transcriptomic data from the cerebral organoids of BPI and healthy control organoids showed downregulation of genes coding for cytoskeletal binding proteins, genes involved in synaptic biology, and genes involved in neurodevelopmental pathways while showing upregulation of immune signaling-related genes. Since gene ontology analyses suggested differences in the endoplasmic reticulum (ER) biology in BPI, we undertook cellular characterization of ER–mitochondria interactions in iPSC-derived neurons using proximity ligation assays. We also used microelectrode array studies in the cerebral organoids to delineate the nature of functional differences in BPI cerebral organoids. To the best of our knowledge, this is the first report of BPI using cerebral organoids and our experiments show how three-dimensional cellular models generated ex vivo from patient iPSCs can be utilized to investigate the neurobiology of psychiatric disorders.

## Methods

### Generation of cerebral organoid from iPSCs

With approval from the Massachusetts General Hospital and the McLean Hospital Institutional Review Boards (IRB), fibroblasts from patients with BPI and matched healthy control individuals who had given informed consent were collected. An experienced team recruited and screened patients, obtained informed consent, collected detailed clinical histories, and isolated fibroblasts through skin punch biopsies. The initial patient recruitment was based on referral from treating psychiatrists for patients between ages of 18 and 65 and diagnosed with bipolar disorder. All the subjects were recruited from McLean Hospital, the primary psychiatric hospital for Harvard Medical School, that has specific treatment units for patients with bipolar disorder and schizophrenia. The initial subject recruitment was based on medical records for patients diagnosed by the treating psychiatrists in the hospital with bipolar disorder. The subject enrollment process included a Structured Clinical Interview for DSM Disorders (SCID) [23], a standard research instrument for ascertaining diagnoses for research studies, and a detailed examination of their clinical records to corroborate the diagnoses and history of treatment response. For healthy control subjects, we selected sex- and age-matched individuals with no history of psychiatric diagnoses or treatment and who did not have any family history of bipolar disorder or schizophrenia. Demographic characteristics of the eight BPI patients and eight healthy control individuals are presented in Additional file 1: Table S1. The fibroblasts were reprogrammed into iPSCs through induction using modified mRNA or with transient transfection with retroviruses using methods described before and validated using standard protocols [24–26] (Additional file 1: Figure S3, Additional file 1: Table S1, Additional files 2 and 3). These iPSCs were differentiated to generate cerebral organoids patterned after the dorsal forebrain [27]. After initially culturing and maintaining the iPSCs in NutriStem media (Stemgent), they were plated on U-bottom plates at a high density to form embryoid bodies (EBs), maintaining them for 5 days in EB formation media with 5 mM Y-27632 and media changed every other day. The EBs generated in the process were re-suspended for 2 days in induction media as per the STEMdiff Cerebral Organoid Kit instructions. For the expansion phase, the EBs were embedded in Matrigel using the droplet hang method on day 7. On day 10, media were switched to maturation media (STEMdiff Cerebral Organoid Maturation Kit) and grown on an orbital shaker in a 37 °C incubator, with media changed every 3–4 days. Ten nanogram per milliliter brain-derived neurotrophic factor (BDNF) was added to the media on day 30 and media changed every 3–4 days thereafter.

### Differentiation of iPSCs to excitatory cortical neurons

iPSCs were cultured on Geltrex (ThermoFisher Scientific, A1413202) in NutriStem hPSC XF Medium (Biological Industries, 01-0005). Confluent iPSC cultures were differentiated into cortical excitatory neurons using standard protocols [26, 28]. The culture media were replenished daily for 7 days using culture media made with 50% N2 medium comprising 485 mL neurobasal medium (Life Technologies, 21103049), 5 mL N-2 supplement (Gibco, 17502001), 5 mL Glutamax (ThermoFisher Scientific, 35050061), and 5 mL penicillin-streptomycin (Gibco 15140122), and 50% B-27 medium comprising 10 mL B-27 supplement (Gibco, 17504044), 480 mL Dulbecco’s modified Eagle medium (DMEM) medium (Sigma-Aldrich, D6421), 5 mL Glutamax (ThermoFisher Scientific, 35050061), and 5 mL penicillin-streptomycin (Gibco 15140122) supplemented with 10 μM SB431542 (Sigma-Aldrich, S4317), 1 μM dorsomorphin (Sigma-Aldrich, P5499), and 100 nM LDN193189 (Sigma-Aldrich, SML0599). On days 8–29, the cells were fed daily with media comprising of 50% N2 and 50% B-27 but without the SMAD inhibitors. For days 30–90, the cultures were replenished twice a week, with Brainphys Neuronal Medium and B-27 supplement.

### Immunohistochemistry—sample preparation

OCT method—Organoids were first fixed for 30 min in 4% paraformaldehyde (PFA) and then rinsed in phosphate-buffered saline (PBS) three times for 5 min each. They were placed in 30% sucrose overnight and embedded in molds in Tissue-Plus O.C.T. Compound (Fisher HealthCare), keeping the samples in dry ice until they were completely frozen. A cryostat was used to section the samples at 20-μm thickness.

Paraffin method—Organoids were fixed for 30 min in 4% PFA and rinsed in PBS three times for 5 min each. They were then dehydrated in a series of ethanol and xylene washes (40 min on 75% ethanol, 40 min on 80% ethanol, 20 min on 95% ethanol, 25 min twice on 100% ethanol, 30 min on 100% ethanol, 30 min twice on xylene, 60 min on xylene, and 60 min twice on paraffin) before they were embedded in paraffin. The embedded samples were left to cool for 2 h, and a microtome was used to section the samples at 20-μm thickness.

### Immunohistochemistry

The slides were rinsed with PBS followed by PBS + 0.1% Triton-X for 10 min, and they were blocked in PBS + 2.5% goat serum and 2.5% donkey serum for 60 min. They were incubated overnight at 4 °C with primary antibodies diluted in PBS. After rinsing the slides with PBS three times for 5 min each, they were incubated at room temperature with secondary antibodies diluted in PBS for 60 min. After rinsing the slides with PBS three times for 5 min each, they were put on a coverslip using the Vectashield Hardset Mounting Medium. Additional file 1: Table S2 lists the antibodies that were used. The PerkinElmer Opera Phoenix High-Content Screening System was used to image the organoid sections at × 20 objective.

### Transcriptome analysis

The Illumina RiboZero TruSeq Stranded Total RNA Library Prep Kit (Illumina) was used to construct the RNA-seq library, and the Illumina NovaSeq6000 platform was used for sequencing in the 100 nt, paired-end configuration. An average of 60 million reads was obtained for each sample. Trimmed reads with Cutadapt were aligned to the reference genome (hg38 UCSC assembly) for gene expression analyses, using TopHat v2.0.14 and Bowtie v2.10 with default parameters and RefSeq annotation (genome-build GRCh38.p9) [29]. Cufflinks v2.2.1 was used to analyze the distribution of alignments, and FPKM (fragments per kilobase of exon model per million reads mapped) values were quantile normalized. Cuffdiff v22.1 was used to perform differential expression testing (Additional file 4) [30, 31]. The false discovery rate (FDR) was 0.05, and sex differences were not considered. Tables 1, 2, and 3 show *q* values representing FDR-adjusted *p* value of the test statistic. RT-PCR was used to validate a number of key relevant genes (Additional file 1: Figure S4).

**Table 1:**
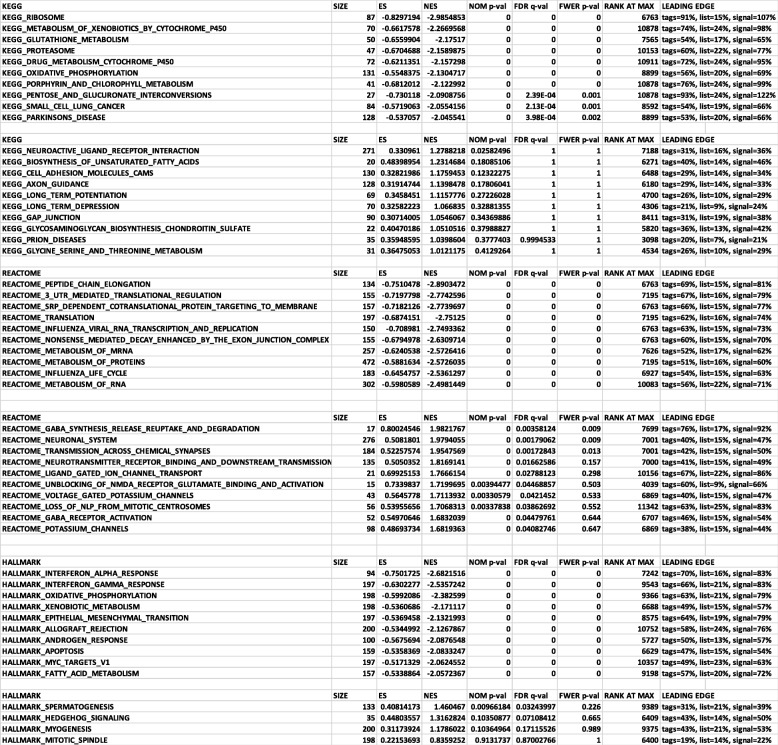
Gene set enrichment analysis (GSEA) analysis

**Table 2:**
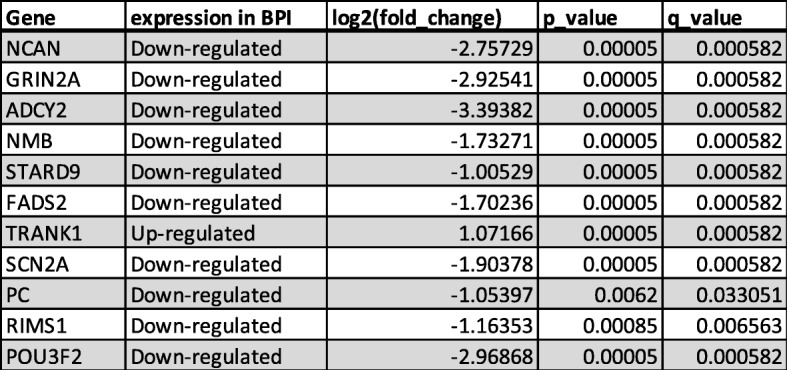
Bipolar disorder GWAS genes that were differentially expressed in BPI cerebral organoids, showing the direction of change compared to healthy control cerebral organoids, fold change, and *p* and *q* values

**Table 3:**
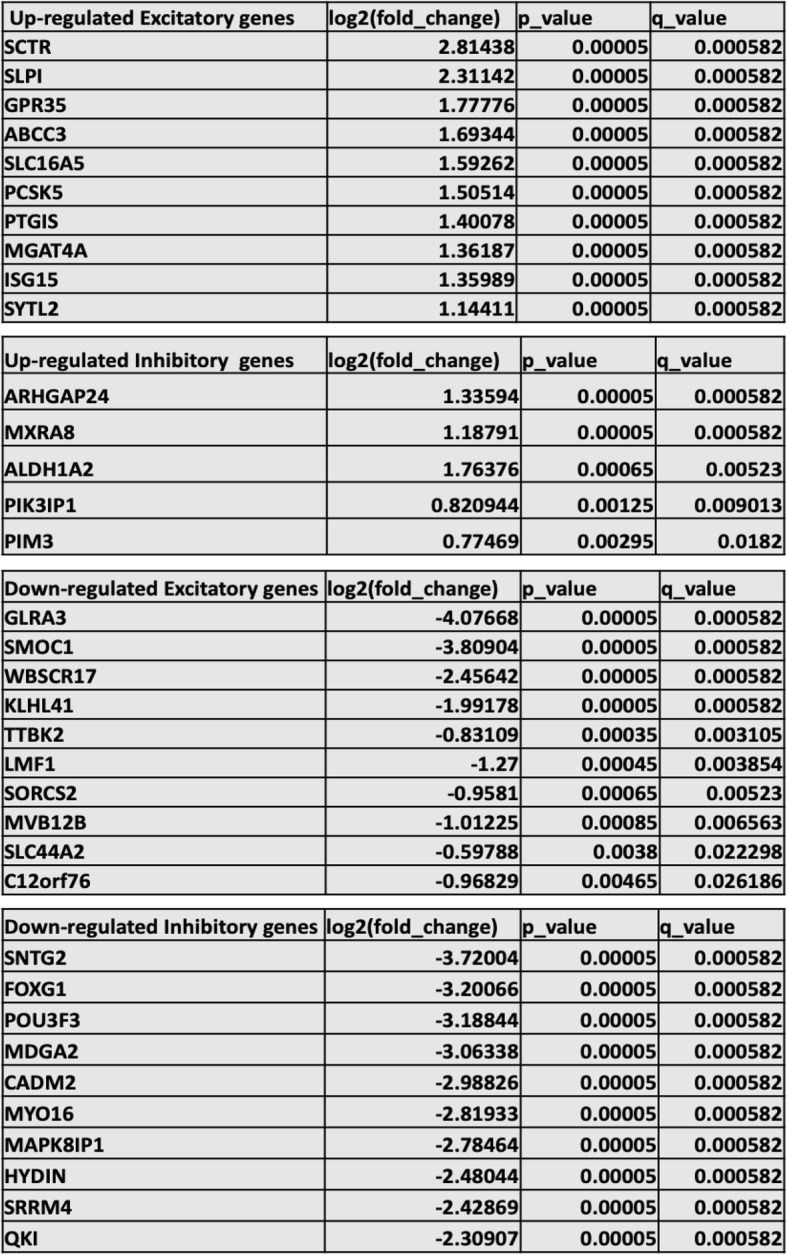
List of top ten significantly upregulated and downregulated genes that are primarily expressed in excitatory and inhibitory neurons, listed according to significance (*p* value)

### Gene ontology and gene set enrichment analyses

Gene ontology (GO) and KEGG analysis was used on all differentially regulated genes with the functional enrichment analysis unit of HOMER v.3 for process, localization, and molecular function [32]. MetaCore+MetaDrug™ version 19.1 build 69600 was used to analyze metabolic processes. The lists depicted in the figures are ones that reached significance (*p* < 0.05). Gene set enrichment analysis (GSEA) was performed with default parameters using the GSEA software for all expressed genes with FPKM values calculated by Cufflinks against the following data sets v6.2: Hallmark, REACTOME, and KEGG [33].

### Protein–protein interaction network generation and analysis

The Search Tool for the Retrieval of Interacting Genes (STRING) (version 11.0; http://www.string-db.org/), a database of known and predicted protein–protein interactions (PPI), was used to construct a PPI network for the differentially expressed genes (DEGs). The interactions in STRING are collated from genomic contexts, co-expression, high-throughput experiments, and reports in the literature, and they include both direct (physical) and indirect (functional) associations [34]. Cytoscape v3.7.1 software was used to visualize and analyze PPI (https://cytoscape.org/), representing PPI networks as graphs with nodes depicting proteins and edges illustrating associated interactions. The Network Analyzer in Cytoscape was used for network analysis, with HUB nodes representing protein nodes of the PPI network with connectivity degree ≥ 10. For the network and GO analyses, the following databases were used: bipolar disorder-associated genes: https://www.med.unc.edu/pgc/pgc-workgroups/bipolar-disorder/; bipolar GWAS genes from Stahl et al. [21]; schizophrenia: http://www.szdb.org/SZDB/score.php; and autism spectrum disorder: https://gene.sfari.org/database/human-gene/.

### Proximity ligation assay

Duolink In Situ PLA Probe kit (Sigma-Aldrich DUO92002, DUO92004) was used for proximity ligation assay (PLA) to quantify interactions between the endoplasmic reticulum and mitochondria in the cortical neurons [35]. Cortical neurons differentiated from iPSCs for 90 days were fixed with 4% PFA for 20 min. The cells underwent washes with PBS thrice after which they were permeabilized with 0.1% Triton-X100 for 15 min. A blocking solution provided in the Duolink In Situ PLA Probe kit was added to each sample. The primary antibodies were diluted in PBS and added to the cultures—VDAC1: 1/100, anti-VDAC1 antibody [20B12AF2] (Abcam, ab14734) and IP3R1: 1/500, anti-IP3 receptor antibody (Abcam, ab5804). The PLA probes were diluted 1:5 in the diluent and incubated in the cultures for 1 h at 37 °C. The Duolink® In Situ Detection Reagent was used for ligation and amplification, followed by a wash with saline sodium citrate (SSC) buffer for 2 min and repeat wash with 0.01x SSC washing buffer for 2 min. Prolong antifade with DAPI was added, and neurons were visualized with high-content imaging at 60x resolution with the PerkinElmer Opera Phoenix High-Content Screening System. The Opera Phoenix and Harmony software (Perkin Elmer) were used to quantify the number of PLA spots.

### Microelectrode array studies

Six-month-old cerebral organoids were attached on MEA 24 well plate that had been coated with 10 μg/ml poly-L-ornithine (Sigma P3655) and 10 μg/ml laminin (Sigma L2020). After 3 months of culture, the cerebral organoids were studied that contained 16 electrodes in each well (Med64, Presto). Spontaneous activity was recorded first, followed by administration of electrical pulse at 0.8 V and with exposure to 30 mM KCl. After each stimulation or KCl exposure, electrical activity was recorded for 60 s. MEA Symphony software was used to analyze the data. To validate the nature of the neuronal electrical activity, recordings were obtained in the presence of 1 mM tetrodotoxin (TTX) (Additional file 1: Figure S3C).

## Results

### Generation and characterization of cerebral organoids

The iPSCs reprogrammed from fibroblasts of patients with BPI and healthy control individuals were differentiated along the telencephalic lineage to generate cerebral organoids [18]. These cerebral organoids, which are patterned after the dorsal forebrain, continued to grow in size during the differentiation process. The average diameter of the cerebral organoids after 1 month was 800 μm to 1 mm, while the average diameter was 2–3 mm after 4 months and 4–5 mm after 7 months (Additional file 1: Figure S1A). We used immunohistochemistry to characterize the cerebral organoids by staining organoid slices with a number of cellular markers. There were no gross differences between cerebral organoids generated from BPI iPSCs compared to those generated from control iPSCs. All cerebral organoids showed expression of the following markers: MAP 2, Ctip2, Satb2, Pax6, TBR2, Cux1, LHX6, glutamine synthetase (GS), GFAP, oligodendrocytes-specific protein/claudin11 (OSP), myelin basic protein (MBP), and IBA1 (Additional file 1: Figure S1B-D). Quantification of neuronal subtypes in organoids from BPI and healthy control lines showed a similar proportion of different cell types in the organoids (Additional file 1: Figure S1E), suggesting consistency in the nature of organoids generated from the different lines.

### Gene expression profiles of cerebral organoids from patients with bipolar disorder and healthy control individuals

**Fig. 1:**
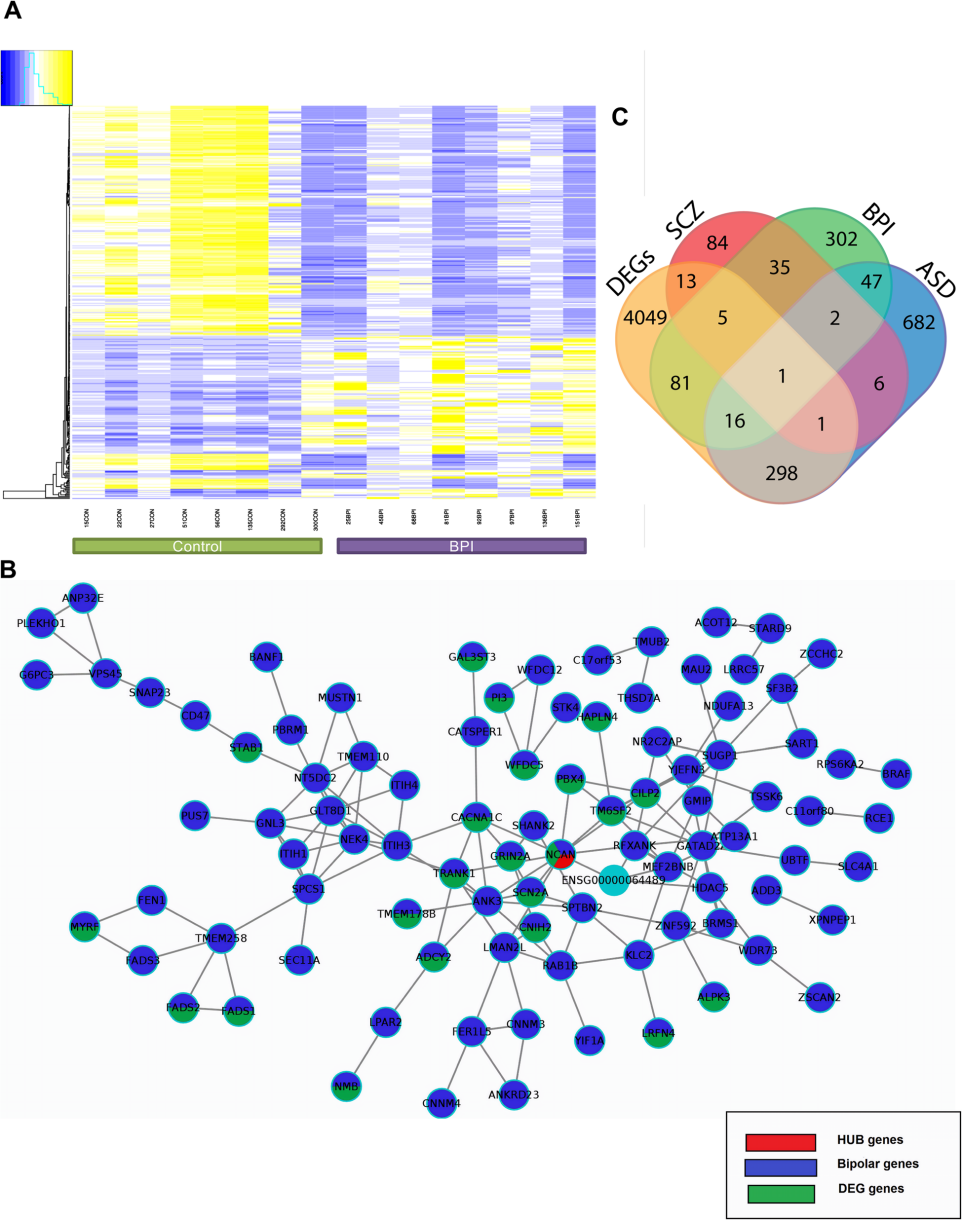
Cerebral organoids generated from human iPSCs. **a** Heatmap for all differentially expressed genes. FPKM values were used with a hierarchical clustering algorithm for gene clustering. **b** Network analysis of DEGs with bipolar disorder-associated genes. **c** Venn diagram showing overlap of DEGs with genes associated with bipolar disorder (BPD), schizophrenia (SCZ), and autism spectrum disorder (ASD)

We collected total RNA sequencing data of 6-month-old cerebral organoids generated from eight BPI and eight healthy control individuals. Sixty million reads were obtained for each sample, and reads were trimmed using Cutadapt and aligned to the reference genome (hg38 UCSC assembly). For gene expression analysis, we used TopHat v2.0.14 and Bowtie v2.10 with default parameters and RefSeq annotation (genome-build GRCh38.p9) [36]. Cufflinks v2.2.1 was used to analyze the distribution of alignments, and FPKM (fragments per kilobase of exon model per million reads mapped) values were quantile normalized. Cuffdiff v22.1 was used to perform differential expression testing. Tables 1, 2, and 3 show *q* values representing the FDR-adjusted *p* value of the test statistic. The total number of DEGs was 4473, out of which 2417 genes were upregulated and 2057 genes were downregulated in BPI. With principal component analysis, we assessed line-to-line and group-to-group variability and found that the gene expression data revealed a group-specific separation between the BPI and control organoids (Additional file 1: Figure S2A). Heatmaps depicting the differentially expressed genes (DEGs) showed a distinct difference in the gene expression pattern in BPI cerebral organoids when compared to healthy control cerebral organoids, for both coding genes and non-coding genes (Fig. 1a, Additional file 1: Figure S2B-C, Additional file 4).

### Gene ontology and gene set enrichment analysis of BPI and control DEGs reveal differences in neurodevelopmental pathways

**Fig. 2:**
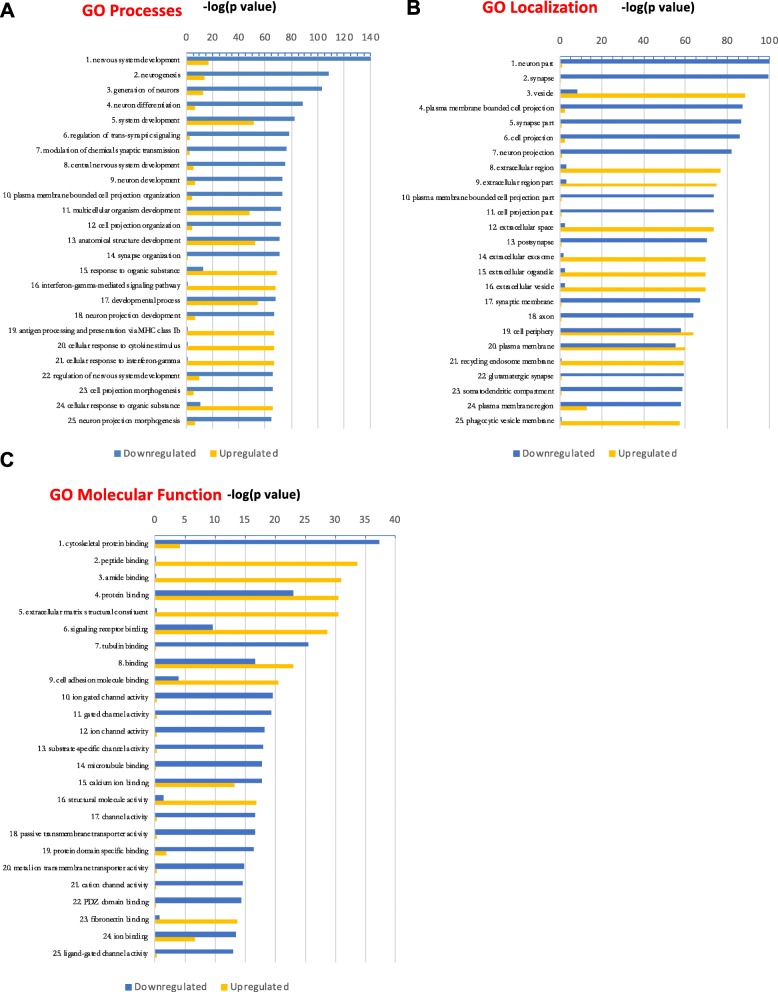
Gene ontology of DEGs in BPI cerebral organoids. **a**–**c** GO analysis of biological processes, localization, and molecular function for DEGs, rank-ordered according to significance (*p* value) for the top 25 hits and depicted as upregulated (yellow) or downregulated (blue) genes

We categorized the DEGs into upregulated and downregulated genes and rank-ordered the top 25 hits according to significance (*p* value) (Fig. 2a–c; Additional file 5). The most significant GO:biological processes that are downregulated in BPI are nervous system development, neurogenesis, generation of neurons, and differentiation of neurons while the most upregulated GO:biological processes in BPI are the IFNγ signaling pathway and antigen processing and presentation of exogenous peptide antigen via major histocompatibility complex (MHC) class Ib (Fig. 2a). GO:localization analysis showed significant downregulation in the synapse, neuron part, and neuronal projection categories in BPI while showing upregulation in the categories of the vesicle and extracellular region (Fig. 2b). We quantified the presynaptic protein Bassoon and the post-synaptic protein Homer in the cerebral organoids from BPI and CON subjects. We found that there was a significant reduction in the levels of Bassoon and Homer in the BPI organoids (Additional file 1: Figure S5). GO:molecular function analysis revealed cytoskeletal binding proteins and ion channel activity to be the categories that are most significantly downregulated in BPI while peptide binding and extracellular matrix structural constituent are the most significantly upregulated categories (Fig. 2c).

We further analyzed the data with gene set enrichment analysis (GSEA), a computational method that allows us to determine if a set of genes can separate two biological categories in a statistically significant manner [33]. GSEA was performed with default parameters using the GSEA software for all expressed genes with FPKM values calculated by Cufflinks against the following data sets v6.2: Hallmark, REACTOME, and KEGG. The pathways that were identified as the most significant involved neuronal function, GABAergic system, glycolysis, and cell adhesion molecules (Table 1). Many of the individual genes in the categories for neuronal function and cell adhesion play important roles in neurodevelopment, and they correspond to pathways that were significant in the GO:localization and GO:molecular function analyses (Fig. 2a–c). In the GO:molecular function analysis, cytoskeletal protein, tubulin binding, and microtubule binding are expressed at lower levels in BPI cerebral organoids, while the GO:localization analysis shows downregulation of genes involved in neurodevelopment processes such as neurogenesis, neuronal projection, and neuronal differentiation. Another gene-enriched pathway in the BPI DEGs is the GABA synthesis, release, uptake, and degradation pathway (Table 1). Most genes in this pathway are downregulated in the BPI cerebral organoids when compared to control organoids. Taken together, these results suggest aberrant neurodevelopmental processes in cerebral organoids from BPI patients. Another gene-enriched pathway in BPI cerebral organoids is the glycolysis pathway (Table 1). This is interesting in the context of clinical studies that show aberrant bioenergetic profiles in the brains of patients with BPI—magnetic resonance spectroscopy (MRS) studies show decreased oxidative phosphorylation resulting in changes in glycolysis and in elevated lactate levels in BPI [37].

### Analysis of protein–protein interaction (PPI) network

STRING was used to construct a PPI network constructed with the DEGs [34] which showed the overlap of the DEGs with genes identified in BPI genetic studies (https://www.med.unc.edu/pgc/pgc-workgroups/bipolar-disorder/), indicating that gene expression differences in the cerebral organoids reflect some of the genetic factors that have been implicated in BPI (Fig. 1b, Table 2). The data have been deposited in NCBI’s Gene Expression Omnibus [38] and are accessible through GEO Series accession number GSE134497 (https://www.ncbi.nlm.nih.gov/geo/query/acc.cgi?acc=GSE134497). We compared the PPI results and found that gene expression patterns in the cerebral organoids mirrored some of the findings in BPI genetic studies. When the DEGs were compared with BPI genetic studies, there were 20 genes that were shared between the two lists (Fig. 1b, Table 2). 36.6% of genes that have genome-wide significance in bipolar disorder [21] were differentially regulated in BPI and control organoids—one of the GWAS genes was upregulated in BPI organoids while 10 GWAS genes were downregulated (Table 2). The GO:localization category that was most significant for the overlapping genes was the membrane region (Fig. 1c, Table 4). The GO:molecular function category that was the most significant in the overlap with BPI-related genes was the “pyruvate carboxylase activity” category, a pathway that plays a pivotal role in the synthesis of glutamate and GABA in the brain (Table 4) [39, 40]. The GO:process category that was the most significant for the overlapping genes was regulation of presynaptic membrane potential (Table 4). This result is interesting in the context of previous studies indicating the presence of presynaptic dysfunction in bipolar disorder [41].

**Table 4:**
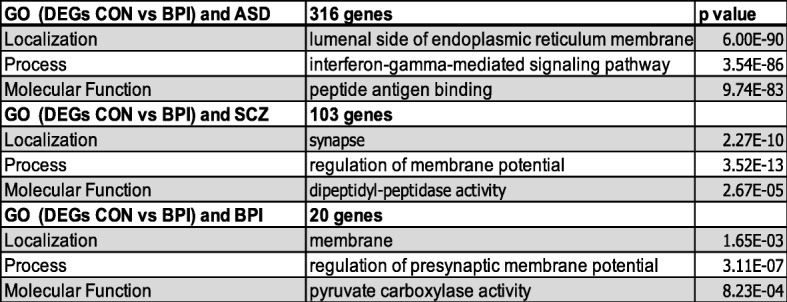
GO analysis of DEGs that overlap with genes associated with BPI, SCZ, and ASD

The central hub gene in the network analysis of the DEGs was neurocan (NCAN), which was significantly downregulated in BPI cerebral organoids (Fig. 1b). NCAN is a proteoglycan that is a component of the neuronal extracellular matrix that is expressed during the modeling and remodeling of neuronal tissue and modulates neural adhesion and migration [42–45]. Genome-wide association studies have implicated common variation in NCAN as a risk for BPI [21, 22, 46, 47]. Functional magnetic resonance imaging (fMRI) studies in humans show that the risk allele affects neural processing and cognitive performance in humans [48]. In human genetic studies, the risk allele for NCAN was significantly associated with the presence of manic episodes [49]. Patients with NCAN risk alleles were also found to have different cortical folding patterns in specific areas of the prefrontal and occipital cortex [50]. In addition, studies in mice showed that loss of NCAN resulted in hyperactivity, increased risk-taking behavior, and impaired pre-pulse inhibition, behaviors that were ameliorated with administration of lithium, which is used to treat BPI [49].

Recent comparative studies of different neuropsychiatric disorders have led to the discovery of shared genetic susceptibility between a number of neuropsychiatric disorders [51]. These differences are reflected in shared neuropathology as well as in polygenic overlap [52]. We compared the DEGs that were significant in our study with schizophrenia-associated genes and autism spectrum disorder (ASD) SFARI genes [53]. The highest overlap with the DEGs was observed with autism-associated genes—316 genes were shared between the DEGs and ASD SFARI genes (Fig. 1c; full list of genes is presented in Additional file 4). In comparing the DEGs overlapping with ASD SFARI genes, the GO:localization category that was the most significant was the lumenal side of the endoplasmic reticulum (ER) membrane (Table 4). The lumenal side of the endoplasmic reticulum (ER) membrane is involved in protein transport, protein modifications, and protein folding with the aid of chaperones [54, 55]. Protein modifications mediated by the lumenal side of ER include N-linked glycosylation, disulfide bond formation and oligomerization [56]. In comparing the overlap of DEGs with ASD-associated genes, the GO process category that was most significant was “interferon-gamma (IFNγ) signaling.” IFNγ has a complex role in mediating immune activation in the central nervous system and is believed to play an important role in the biology of ASD [57, 58]. While peripheral cytokine patterns have been noted to be different in BPI, there have been conflicting reports on the role of IFNγ in BPI [59, 60]. When GO:molecular function categories were analyzed in relation to the overlap with ASD SFARI genes, the “peptide antigen binding” category was found to be the most significant, which refers to the binding of peptides to MHC molecules. These results suggest that IFNγ signaling and the immune response may be relevant in the context of neurodevelopmental processes in BPI [61, 62].

GO:localization analysis of genes shared between the DEGs and SCZ-associated genes showed the synapse to be the most significant category (Table 4). The GO:biological process category that was most significant in the overlap with SCZ-related genes was regulation of membrane potential (Table 4). GO:molecular function analysis of the DEG overlap with SCZ-associated genes showed dipeptidyl-peptidase activity as the most significant category (Table 4).

### Mitochondria-associated endoplasmic reticulum membranes in cortical neurons

**Fig. 3:**
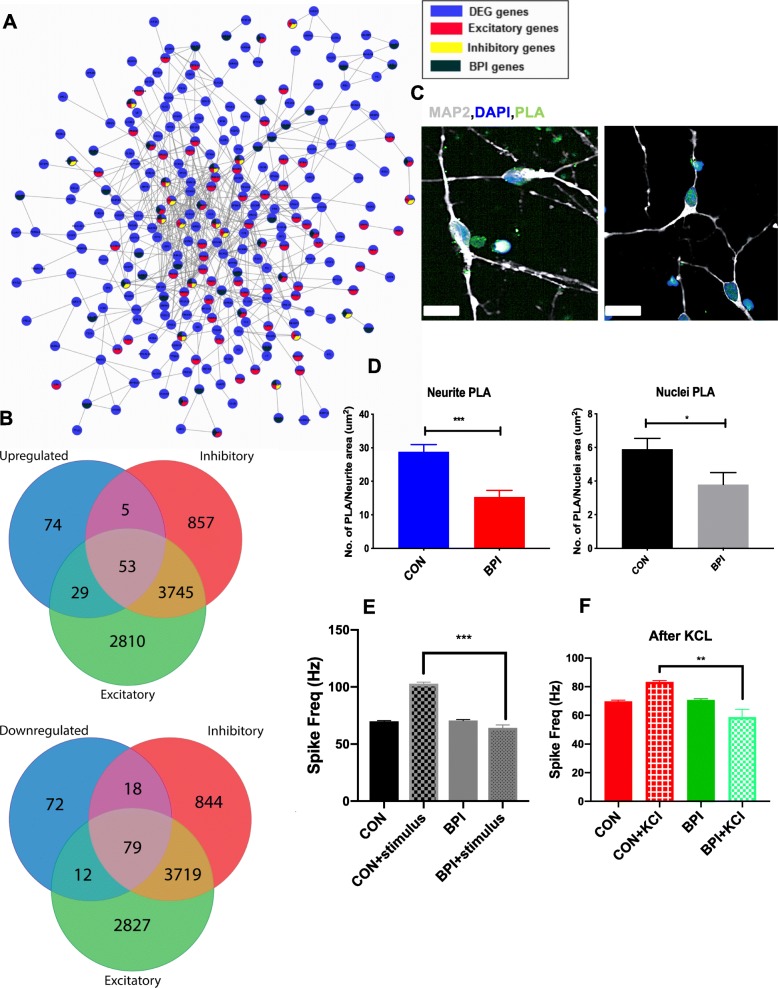
Functional characterization of cerebral organoids. **a** Co-expression patterns of DEGs with genes expressed in excitatory and inhibitory neurons. **b** Overlap of upregulated and downregulated DEGs with genes expressed in excitatory and inhibitory neurons. **c**, **d** Visualization and quantification of ER–mitochondria-associated membranes in cortical neurons, using PLA assays conducted with antibodies against VDA1 and IP3R. PLA spots indicting physical association between VDAC1 and IP3R are shown in green, neurites are stained with MAP 2 (white), and nuclei are stained with DAPI (blue) (× 60 magnification; scale bar = 20 μm). The mean numbers of PLA spots were calculated for the nuclear area and the neurites. Values are shown as mean ± SEM. Unpaired Student *t* test with Welch’s correction: ***p = 0.0004 and *p = 0.04. Eight CON lines were compared 8 BPI lines for this analysis. **e** Quantification of raster plot data collected before and after electrical stimulation with 0.8 V. **f** Quantification of raster plot data collected after depolarization with 30 mM KCl. Data was collected for four CON and four BPI iPSC organoids, with two technical replicates in each condition. Values are shown as mean ± SEM. Unpaired Student *t* test with Welch’s correction: ***p < 0.001 and **p = 0.0037

In the gene ontology analyses, the upregulated ER GO terms are the extracellular region, part, and space while the downregulated ER-related GO terms are plasma membrane-bounded cell membrane, part, region, cell projection organization, and passive transmembrane transporter activity. The ER and mitochondria make physical contact and facilitate Ca^2+^ exchange between the two organelles [63]. The ER–mitochondria contact sites, which are called mitochondria-associated endoplasmic reticulum membranes (MAMs), play crucial roles in calcium homeostasis, phospholipid exchange, intracellular trafficking, ER stress and unfolded proteins, autophagy, and inflammation [63]. To examine the MAM biology in the neuronal context, we performed the proximity ligation assay (PLA) in cortical excitatory neurons generated from iPSCs of the BPI and control individuals. When we quantified and compared MAM structures in BPI and control neurons, we found that MAM was significantly reduced in BPI patient-derived neurons when compared to neurons from healthy control individuals (Fig. 3c, d.). This difference was observed not only in the perinuclear region but also in the neurites. The MAM quantification studies provide support for the hypothesis that ER–mitochondria interaction may be compromised in bipolar disorder, resulting in dysfunction of the fundamental cellular processes [64].

### Functional studies of cerebral organoids with microelectrode arrays

To interrogate the roles of the BPI DEGs involved in synaptic transmission, we built a PPI network from the neural subtype transcriptome [65] and BPI-associated genes (Fig. 3a, Table 3). We classified the DEGs as BPI-associated [21] excitatory or inhibitory genes and then sub-classified them into upregulated and downregulated genes. Out of a total 4474 DEGs, 342 were classified into excitatory and inhibitory subtypes, which included 161 upregulated and 181 downregulated DEGs. Among the 87 upregulated genes, 29 were expressed in excitatory neurons, 5 were expressed in inhibitory neurons, and 53 were expressed in both excitatory and inhibitory neurons (Fig. 3b, Additional file 1: Tables S3-S5). Among the 109 downregulated genes, 12 were expressed in excitatory neurons, 18 were expressed in inhibitory neurons, and 79 were expressed in both excitatory and inhibitory neurons (Fig. 3b, Additional file 1: Tables S3-S5). These data, along with the results of the GO and GSEA analyses, suggest that there may be differences in the balance of excitatory and inhibitory activity in BPI. In order to investigate whether these gene expression differences may be accompanied by differences in synaptic transmission, we used a microelectrode array (MEA) system to evaluate functional properties of neurons that comprise the cerebral organoids [66]. We initially tested 6-month-old and 9-month-old organoids and found that no electrical activity could be recorded in 6-month-old organoids, but regular and consistent activity could be recorded in 9-month-old organoids. When we measured the spontaneous activity of 9-month-old cerebral organoids under normal culture conditions, we found there were no differences in the spontaneous firing rate between in cerebral organoids from BPI and healthy control individuals (Fig. 3e, Additional file 1: Figure S5C). We then measured the neuronal firing rate in the presence of electrical stimulation at 0.8 V. Cerebral organoids from healthy control individuals exhibited a significant increase in spike frequency in response to the electrical stimulus while BPI cerebral organoids showed no such increase compared to their baseline spike frequency (Fig. 3e and Additional file 1: Figure S1D). We also measured spike frequency in response to neuronal depolarization with 30 mM potassium chloride (KCl). Cerebral organoids from healthy control individuals again exhibited a significant increase in spike frequency in response to KCl while BPI cerebral organoids showed no such increase (Fig. 3f and Additional file 1: Figure S1C). Hence, in the MEA experiments, BPI cerebral organoids show normal electrical activity at baseline but exhibit an aberrant diminished response to electrical stimulation and depolarization when compared to cerebral organoids from healthy control individuals.

## Discussion

The study of neuropsychiatric disorders such as BPI has historically been hindered by the lack of access to the disease-relevant tissue from patients, but recent advances in the ability to generate iPSCs from somatic cells provide new avenues to study disease biology using stem cell-based ex vivo cellular models [67]. iPSCs can be readily differentiated to a wide variety of neuronal and glial cell types, enabling the study of cell types that are relevant to disease biology [26, 68–73]. While the use of iPSC-based cellular models to study the neurobiology of BPI has been limited to the use of two-dimensional neuronal cultures, methods to grow brain organoids provide ways to study the disease biology in complex three-dimensional structures that comprise a large number of cell types [74, 75].

Here, we interrogated the neurobiology of BPI using cerebral organoids differentiated from iPSCs of patients with BPI and healthy individuals. We carried out RNA-seq experiments with these cerebral organoids and identified disease-specific differential regulation of genes as well as non-coding RNA. Analysis of the differentially regulated genes points to specific pathways that may be aberrant in BPI, including pathways involved in cell adhesion, neurodevelopment, and synaptic biology as well as genes involved in immune signaling. It is worth noting that there were significant differences in disease-specific expression of non-coding RNA as well (Fig. 1a, Additional file 1, Figure S2B, Additional file 4). There is a growing recognition of important roles for non-coding RNA in psychiatric disorders, including bipolar disorder, though the exact nature of these roles has yet to be delineated [76, 77]. Previous studies with neuroimaging, postmortem brains, and patient-derived cells that have explored the disease biology of BPI have led to results indicating deficits in different neural circuits, reduced glial populations in the prefrontal cortex, differences in the metabolic pathways in the presence of stress, and abnormalities in neuronal and calcium signaling [14, 15, 78–81]. When evaluating results from neuroimaging studies, postmortem tissue, and investigation of primary cells, it is often difficult to delineate the contribution of genetic factors from confounding effects due to medications, non-prescription drugs, stress, environmental effects, or downstream effects of the disease process. Since reprogramming of somatic cells to iPSCs results in the erasure of epigenetic markers, ex vivo cellular models generated from patient iPSCs enable the study of contributions of the complex genetic backgrounds to the disease biology [82].

GO analyses based on the RNA-seq data reported here show that differences in pathways involved in neurodevelopment, neurogenesis, and neuronal differentiation of neurons may result from the underlying complex genetics of BPI. Similarly, the GO and GSEA data implicate differences in BPI in processes involving the IFNγ signaling pathway, cytoskeletal binding proteins, cell adhesion molecules, LPS pathway, and GABAergic systems. The findings of immune system-related pathways as important in these ex vivo studies, including antigen processing via MHC, in the absence of any exogenous infectious or immune exposure in the experimental design is interesting since the MHC locus is one of the most significant loci for schizophrenia and also implicated in bipolar disorder [62, 83]. We find it very interesting that the network analysis of our transcriptomic study and genes associated with BPI led to the identification of NCAN as a central hub for the differentially expressed genes. GWAS data show that NCAN is one of the common variants that are significantly associated with BPI risk [21, 22, 46, 47]. NCAN is a proteoglycan that is involved in cell adhesion and neuronal migration, processes that are pivotal during neurodevelopment [42–45, 84]. Our findings are relevant in the context of the hypotheses and previous evidence for the neurodevelopmental roots of the disease biology of BPI [13, 85].

The RNA-seq data from BPI cerebral organoids in our study showed significant differences in genes involved in modulating the balance between excitatory and inhibitory activity in the brain. To explore this further, we undertook functional studies of the cerebral organoids using MEAs. Measurement of spike frequency in the cerebral organoids showed no differences in baseline electrical activity between BPI and control organoids. Cerebral organoids showed a characteristic increase in spike frequency in response to electrical stimulation or KCl depolarization, but this increase was not present in BPI organoids. These findings are interesting in light of studies of long-range connectivity in cortical areas of BPI patients that showed a significant reduction in gamma coherence in response to target stimuli when compared to the response in healthy individuals [36]. In addition, experiments in animal models and neuronal cultures have shown that mood stabilizers used in the treatment of BPI modulate a number of cellular processes that are involved in excitation/inhibition (E/I) balance [86].

In describing our findings, we want to note some drawbacks and limitations in the use of human iPSCs to study the neurobiology of psychiatric disorders such as BPI. While this is the largest number of iPSC lines that have been used for the study of BPI cerebral organoids, the sample size is still small in the context of the study of complex psychiatric disorder. In addition, while two cerebral organoids were used for each line, the fact that one iPSC clone was used for each subject raises the possibility of clone-specific differences. However, reprogramming with mRNA has been shown to result in reduced clonal variation [87]. We also note that the cerebral organoids that we have generated reflect previous findings that whole-brain cerebral organoids generated from iPSCs reproducibly generate cell diversity seen in the cerebral cortex, but they do not show clear cellular organization [19]. One of the BPI patients had a diagnosis of Klinefelter’s syndrome, but there were no obvious differences in the transcriptomic profile of this patient compared to the other BPI patients. BPI is determined by numerous factors, none of which contributes more than a modest amount to risk and expression of illness. This became evident from genetic studies and is now becoming clear from cell-organ biology studies. This raises the question of whether BPI results from multiple genetic and environmental hits at the cell and organ level. It is interesting to speculate whether specific targets can be identified from the genetic and cell biology studies that can be harnessed to modulate the disease biology. BPI manifests itself fully in late adolescence and early adulthood while cerebral organoids investigated in this report are aged 6–9 months. However, numerous studies point to strong neurodevelopmental contributions to the disease biology in BPI, including anomalies occurring in utero. Certain facets of the neurobiology of disease, especially those related to neurodevelopment, may be tractable to interrogation in iPSC-derived cellular models. We also note that our experiments do not take into account environmental factors that may impinge on the genetic background resulting in disease onset. There are possible ways to use specific perturbations in in vitro cell culture models to simulate gene-environment interactions to reveal cellular pathways relevant to disease biology [80, 88]. The new possibilities of generating ex vivo two-dimensional and three-dimensional cellular models from patient-derived iPSCs provide a valuable method for interrogating the disease biology of neuropsychiatric disorders and to identify cellular processes and molecular targets that can be targeted for therapeutic applications.

## Conclusions

We provide the first comprehensive study of comparing cerebral organoids generated from BPI patients and healthy individuals. We delineate transcriptomic differences in specific pathways, including downregulation of genes involved in cell adhesion, neurodevelopment, and synaptic biology in bipolar disorder along with the upregulation of genes involved in immune signaling. In a network analysis of the differentially regulated genes, the central hub was NCAN, which is located in a locus with genome-wide significant association in BPI. Gene ontology analyses and cellular studies pointed to deficits related to endoplasmic reticulum biology in BPI. Functional studies with microelectrode arrays revealed normal baseline neuronal firing patterns but specific deficits in response to stimulation and depolarization in BPI cerebral organoids. Our results show the power and utility of using patient-derived iPSCs to generate ex vivo three-dimensional cellular models to interrogate the disease biology of neuropsychiatric disorders.

## Supplementary information

**Additional file 1.** Contains supplementary figures and tables.

**Additional file 2.** Karyotyping data for iPSC lines.

**Additional file 3.** Marker analysis for iPSC lines.

**Additional file 4.** Cuffdiff analysis of bulk RNA seq, and data presented in Fig. 1b, Table 2, Table 3 and Table 4.

**Additional file 5.** Gene ontology analysis.

## Data Availability

The datasets generated during the current study have been deposited in NCBI’s Gene Expression Omnibus [38] and are accessible through GEO Series accession number GSE134497: https://www.ncbi.nlm.nih.gov/geo/query/acc.cgi?acc=GSE134497. Analytical tools used in this study are cited within the “Methods” section.
