# Supplementary material for: Transcriptome analysis and functional characterization of cerebral organoids in bipolar disorder
Source: Genome Med. 2020 Apr 19;12:34. doi: 10.1186/s13073-020-00733-6 (PMC7168850; doi:10.1186/s13073-020-00733-6)
Supplement: Supplementary file 1 — Additional file 1. Contains supplementary figures and tables. [file 13073_2020_733_MOESM1_ESM.docx]

**
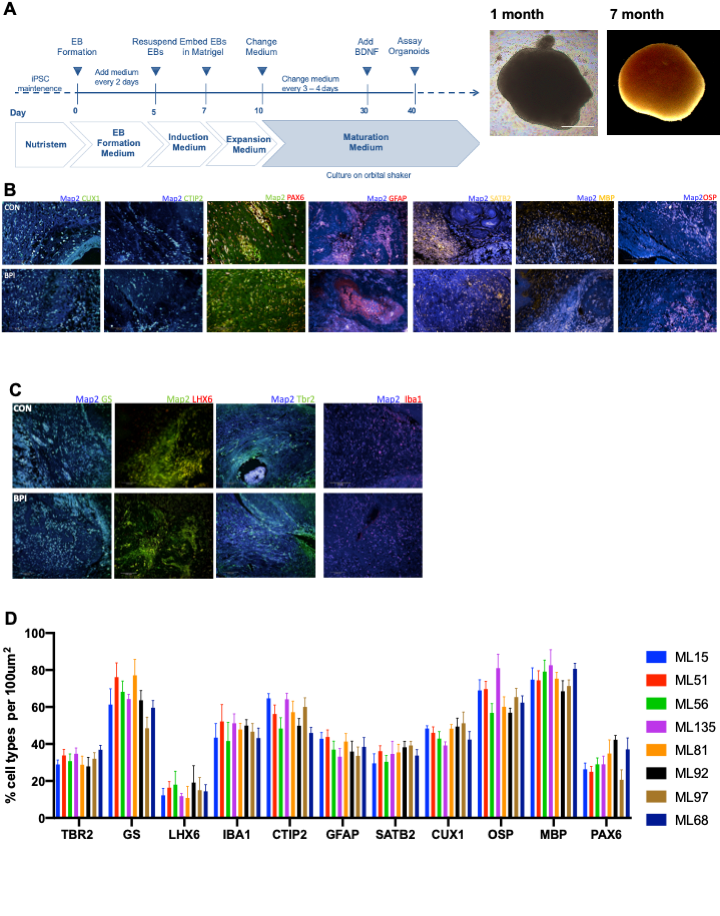
**

**Supplementary Figure 1: Differentiation and characterization of cerebral organoids from human iPSCs**. A. Protocol used for differentiation of cerebral organoids from human iPSCs. Phase-contrast images of cerebral organoids at different stages of development are shown. Scale bar: 250 μm for image of 1 month old organoid and 1 mm for image of and 7 month old organoid. B. Representative images of immunohistochemistry of cerebral organoid slices from BPI and CON organoids. C. Quantification of cell types, where ML15,51,56,and 135 represent CON lines and ML81, 92,97, 68 are BPI lines.

**
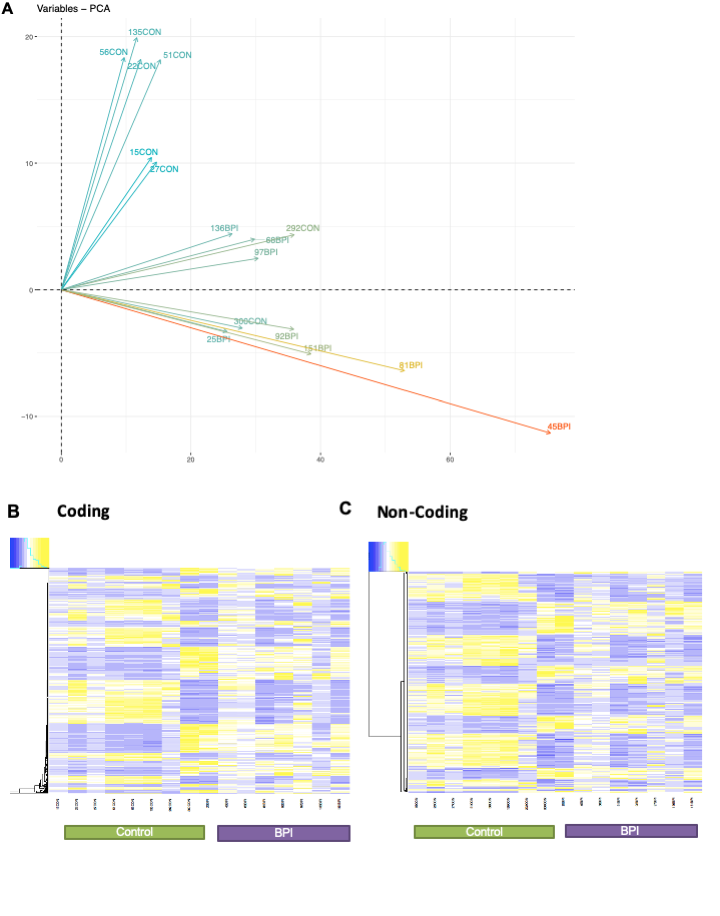
Supplementary Figure 2:**A. Principal component analysis (PCA) to show the distribution of CON and BPI iPSC line generated organoid population. B.Heatmaps for coding and non-coding proteins.


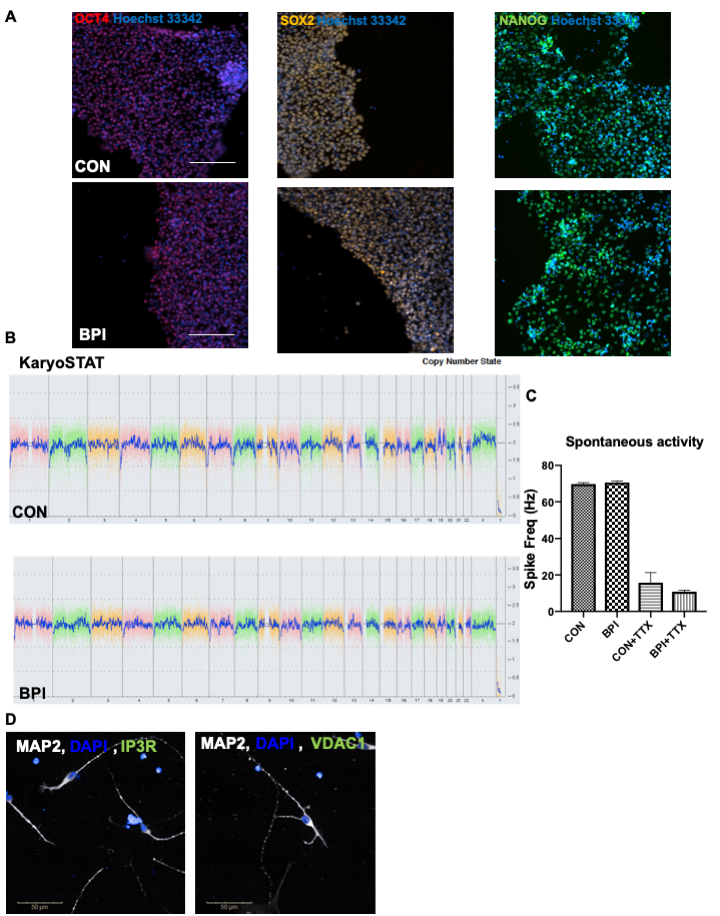


**Supplementary Figure 3.** A. Representative images of immunocytochemistry of CON and BPI iPSC lines for Nanog (green), Oct4 (red), SOX2 (yellow) and Hoechst (blue). Scale bar: 50μm. This characterization has been done for all 16 iPSC lines and data is available on request. B. Representative images of the whole genome view of one CON line and one BPI line. The whole genome view displays all somatic and sex chromosomes in one frame with high level copy number. The smooth signal plot (right y-axis) is the smoothing of the log2 ratios which depict the signal intensities of probes on the microarray. A value of 2 represents a normal copy number state (CN = 2). A value of 3 represents chromosomal gain (CN = 3). A value of 1 represents a chromosomal loss (CN = 1). The pink, green and yellow colors indicate the raw signal for each individual chromosome probe, while the blue signal represents the normalized probe signal which is used to identify copy number and aberrations (if any). C. Spontaneous activity of CON and BPI cerebral organoids with and without TTX 1mM. D. Negative control for the PLA assay demonstrated that using only one primary antibody did not lead to green fluorescence.

**Supplementary Figure 4: RT-PCR validation**. **A-F.** Graphs show relative gene expression in cerebral organoids compared to a control iPSC line assessed via quantitative PCR (qPCR). Values are median±IQR. Mann–Whitney U-test was performed. The significant differences between CON and BPI groups is shown as ***p <0.001. The assay was performed in seven CON and seven BPI lines, each with three replicates.


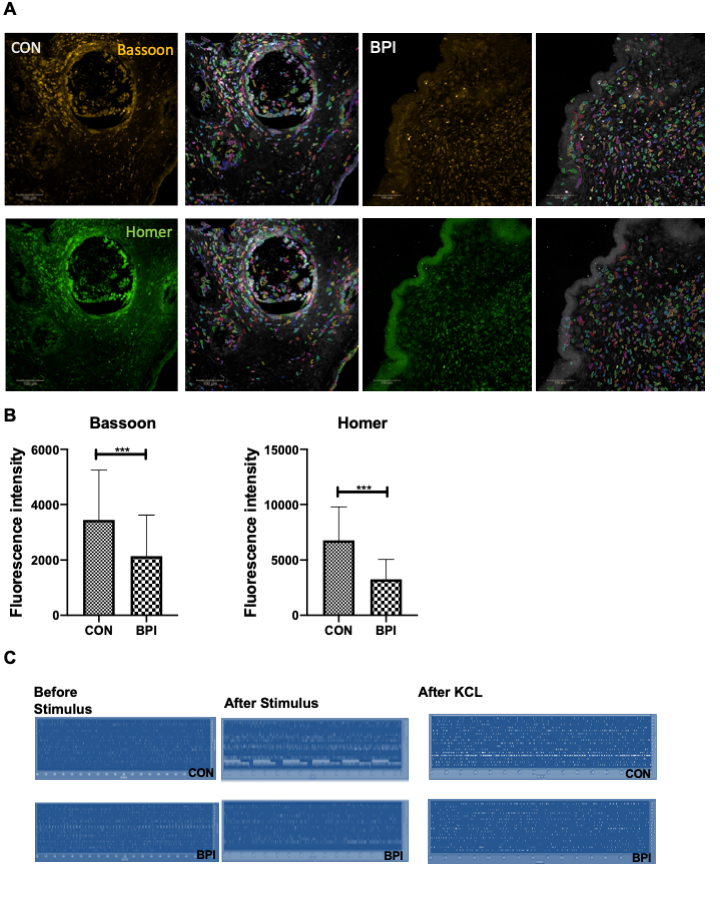


**Supplementary Figure 5: Synaptic markers in cerebral organoids**. **A.** Representative immunocytochemistry images of CON and BPI organoids with pre-synaptic Marker Bassoon and post-synaptic marker Homer. **B.** Quantification of Bassoon and Homer in CON and BPI organoids. Values are mean±SD. Mann–Whitney U-test was performed. The significant differences between CON and BPI groups is shown as ***p <0.001. Scale bar: 100μm. **C**. Representative raster plot recording before and after electrical stimulation, after depolarization with 30 mM KCl, in CON and BPI organoids.


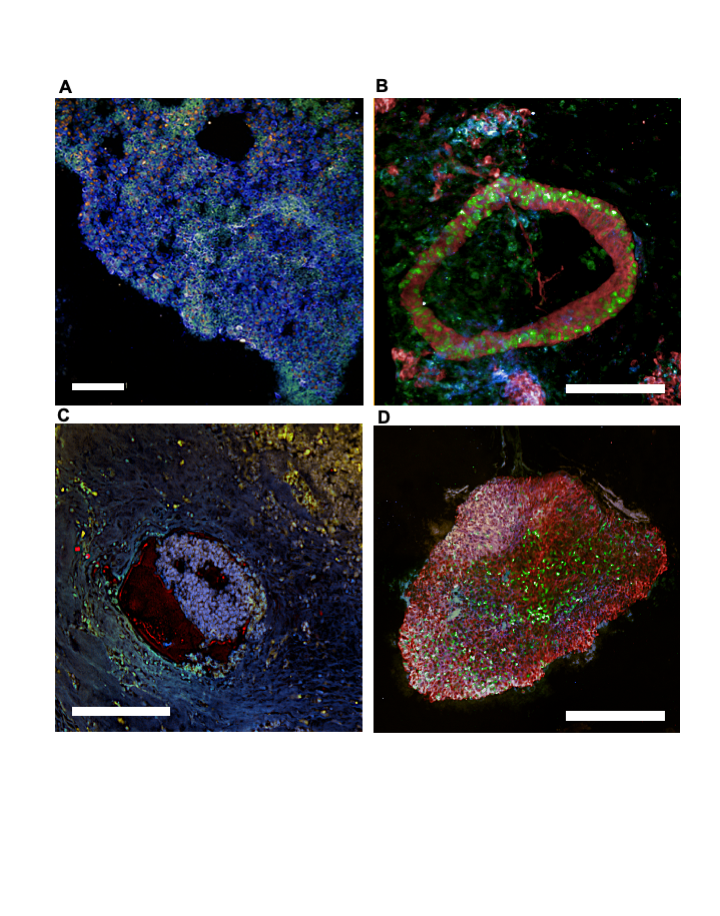


**Supplementary Figure 6: Representative high-magnification images of organoids** stained with the indicated markers. A. MAP2 (blue), GFAP (green), GAD67 (yellow), CUTL1 (red). B. MAP2 (red), Ctip2 (green), Satb2 (yellow), Tbr2 (blue). C. Iba1 (red), glutamine synthetase (green), GAD67 (yellow), MAP2 (blue). Scale bar:100μm. D. MAP2 (red), Ctip2 (green), Satb2 (yellow), Tbr2 (blue). Scale bar:500μm.

**Supplementary Table 1: Patient details for each iPSC line generated.**

**Supplementary Table 2: Primary antibodies.**

**Supplementary Table 3: Downregulated Excitatory genes**

**Supplementary Table 4: Downregulated Inhibitory genes**

**Supplementary Table 5: Upregulated Excitatory genes**
