# Supplementary material for: Transcriptome analysis and functional characterization of cerebral organoids in bipolar disorder
Source: Genome Med. 2020 Apr 19;12:34. doi: 10.1186/s13073-020-00733-6 (PMC7168850; doi:10.1186/s13073-020-00733-6)
Supplement: Supplementary file 2 — Additional file 2. Karyotyping data for iPSC lines. [file 13073_2020_733_MOESM2_ESM.pdf]

KaryoStat™ Results: CUB12899-12

1. KaryoStat™ analysis of CUB12899-12 revealed the sample originated from a male individual
2. No chromosomal aberrations were found when comparing against the reference dataset (Figure 21)

Copy Number State

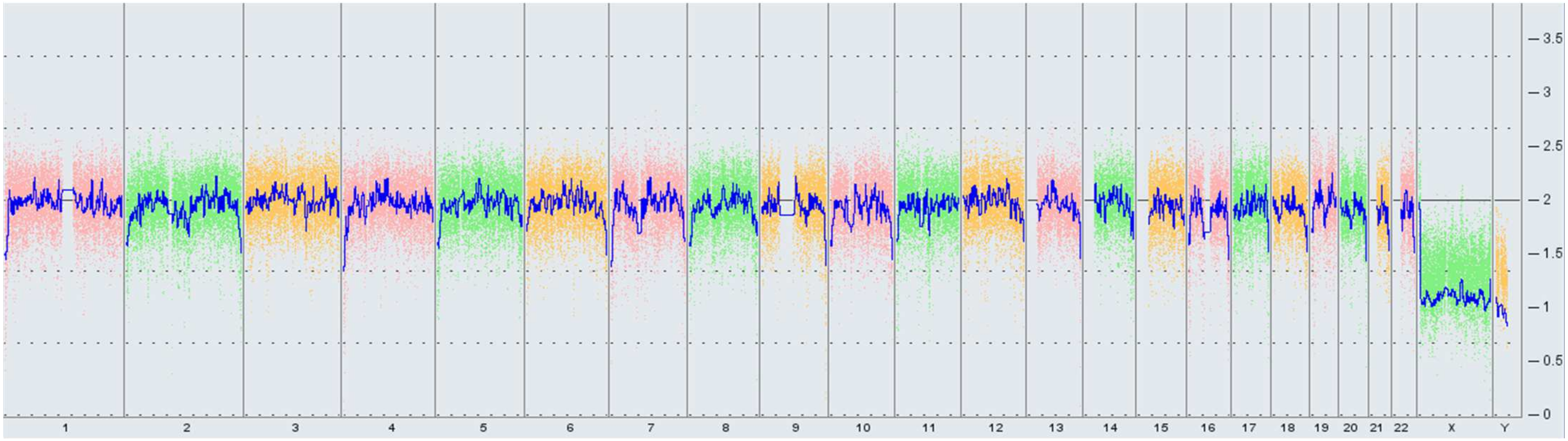

Figure 21: Whole genome view. The whole genome view displays all somatic and sex chromosomes in one frame with high level copy number. The smooth signal plot (right y-axis) is the smoothing of the log2 ratios which depict the signal intensities of probes on the microarray. A value of 2 represents a normal copy number state (CN = 2). A value of 3 represents chromosomal gain (CN = 3). A value of 1 represents a chromosomal loss (CN = 1). The pink, green and yellow colors indicate the raw signal for each individual chromosome probe, while the blue signal represents the normalized probe signal which is used to identify copy number and aberrations (if any).

Disclaimer: This assay was conducted solely for the listed investigator/institution. The results of this assay are for research use only.

ThermoFisher SCIENTIFIC

KaryoStat™ Results: CUB12899-5

1. KaryoStat™ analysis of CUB12899-5 revealed the sample originated from a male individual
2. Chromosomal aberrations were observed when comparing to the reference set (Table 2, Figure 1)
3. A supplemental document with detailed information on the aberration will be provided to the Client

Chromosome	Type	Cytoband Start	CN State	Size (kbp)
15	Loss	q13.2	1	1,348

Table 2: KaryoStat™ analysis. Chromosomal aberrations are indicated in the table shown**.** See supplemental data for more details.

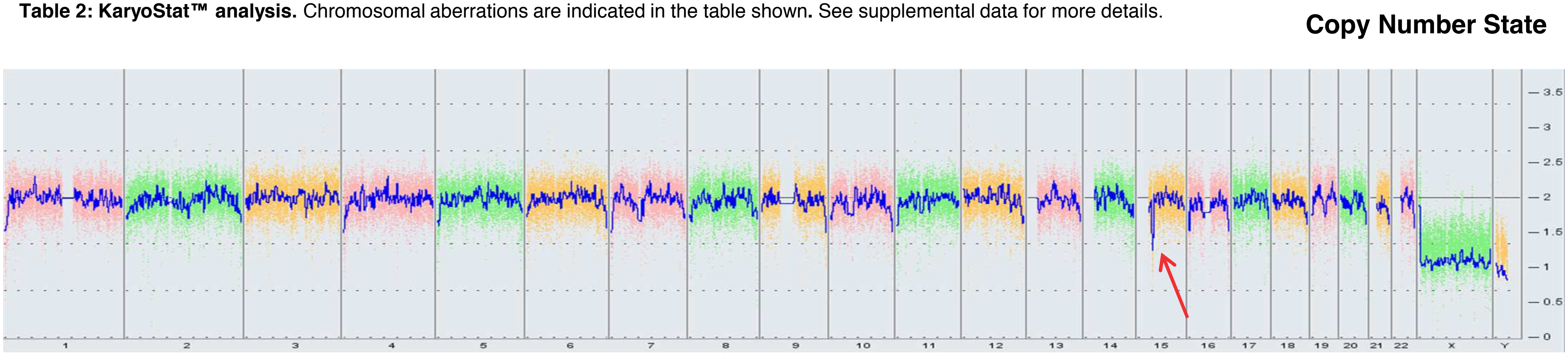

Figure 1: Whole genome view. The whole genome view displays all somatic and sex chromosomes in one frame with high level copy number. The smooth signal plot (right y-axis) is the smoothing of the log2 ratios which depict the signal intensities of probes on the microarray. A value of 2 represents a normal copy number state (CN = 2). A value of 3 represents chromosomal gain (CN = 3). A value of 1 represents a chromosomal loss (CN = 1). The pink, green and yellow colors indicate the raw signal for each individual chromosome probe, while the blue signal represents the normalized probe signal which is used to identify copy number and aberrations (if any). The whole genome view analysis revealed chromosomal aberrations indicated by the red arrow.

Disclaimer: This assay was conducted solely for the listed investigator/institution. The results of this assay are for research use only.

ThermoFisher SCIENTIFIC

KaryoStat™ Results: CUB12899-6

1. KaryoStat™ analysis of CUB12899-6 revealed the sample originated from a male individual
2. No chromosomal aberrations were found when comparing against the reference dataset (Figure 2)

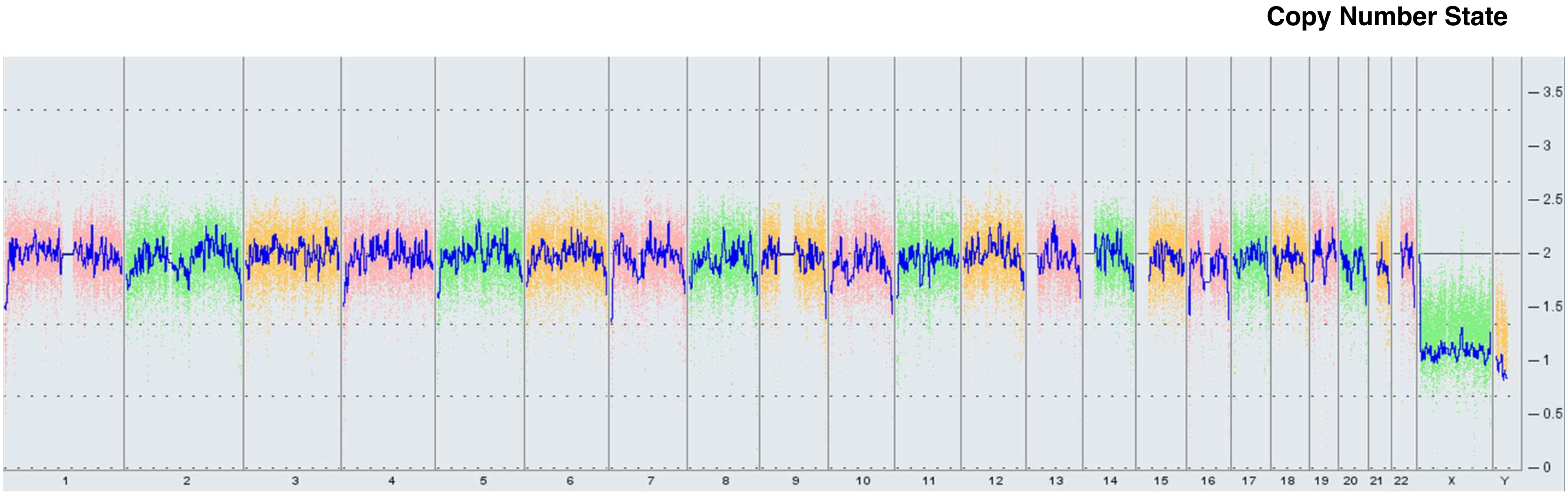

Figure 2: Whole genome view. The whole genome view displays all somatic and sex chromosomes in one frame with high level copy number. The smooth signal plot (right y-axis) is the smoothing of the log2 ratios which depict the signal intensities of probes on the microarray. A value of 2 represents a normal copy number state (CN = 2). A value of 3 represents chromosomal gain (CN = 3). A value of 1 represents a chromosomal loss (CN = 1). The pink, green and yellow colors indicate the raw signal for each individual chromosome probe, while the blue signal represents the normalized probe signal which is used to identify copy number and aberrations (if any).

Disclaimer: This assay was conducted solely for the listed investigator/institution. The results of this assay are for research use only.

KaryoStat™ Results: CUB12899-24

1. KaryoStat™ analysis of CUB12899-24 revealed the sample originated from a male individual
2. No chromosomal aberrations were found when comparing against the reference dataset (Figure 14)

Copy Number State

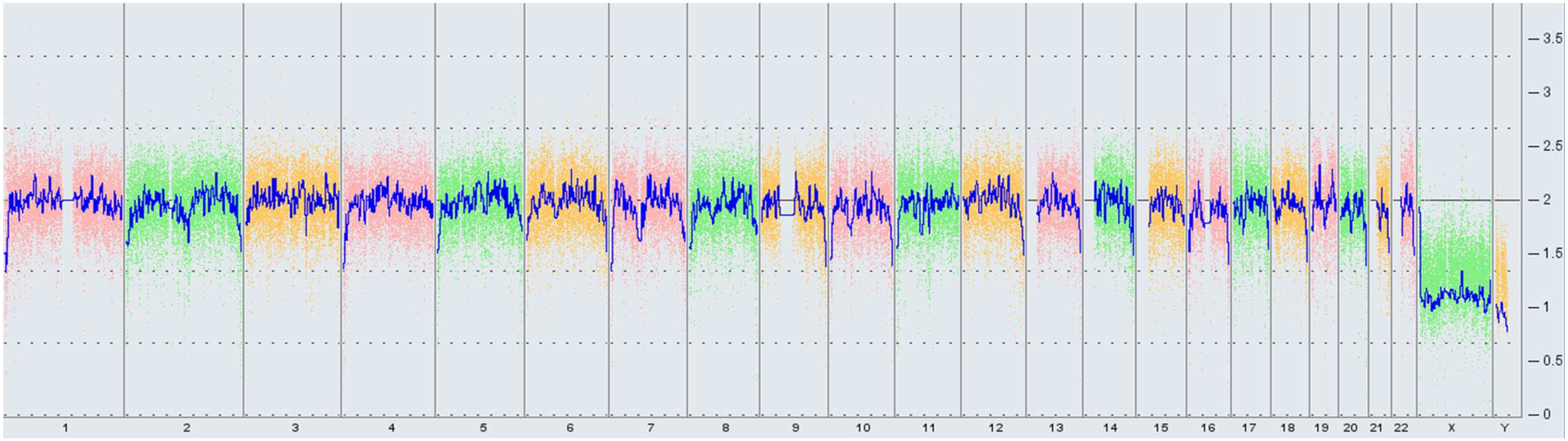

Figure 14: Whole genome view. The whole genome view displays all somatic and sex chromosomes in one frame with high level copy number. The smooth signal plot (right y-axis) is the smoothing of the log2 ratios which depict the signal intensities of probes on the microarray. A value of 2 represents a normal copy number state (CN = 2). A value of 3 represents chromosomal gain (CN = 3). A value of 1 represents a chromosomal loss (CN = 1). The pink, green and yellow colors indicate the raw signal for each individual chromosome probe, while the blue signal represents the normalized probe signal which is used to identify copy number and aberrations (if any).

Disclaimer: This assay was conducted solely for the listed investigator/institution. The results of this assay are for research use only.

Thermo Fisher SCIENTIFIC

KaryoStat™ Results: CUB12899-25

1. KaryoStat™ analysis of CUB12899-25 revealed the sample originated from a female individual
2. No chromosomal aberrations were found when comparing against the reference dataset (Figure 15)

Copy Number State

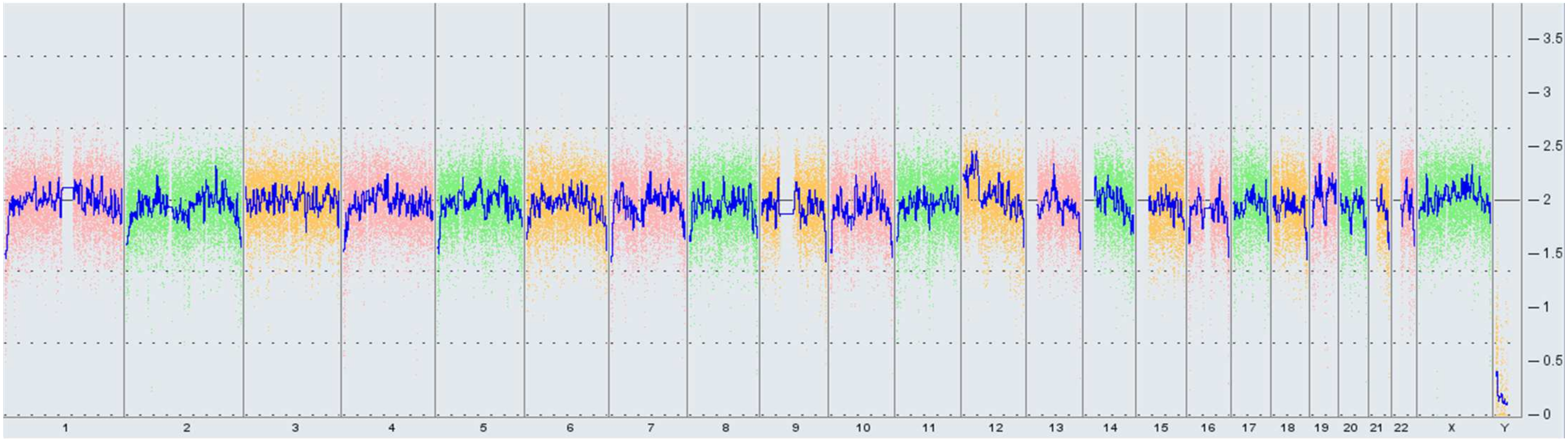

Figure 15: Whole genome view. The whole genome view displays all somatic and sex chromosomes in one frame with high level copy number. The smooth signal plot (right y-axis) is the smoothing of the log2 ratios which depict the signal intensities of probes on the microarray. A value of 2 represents a normal copy number state (CN = 2). A value of 3 represents chromosomal gain (CN = 3). A value of 1 represents a chromosomal loss (CN = 1). The pink, green and yellow colors indicate the raw signal for each individual chromosome probe, while the blue signal represents the normalized probe signal which is used to identify copy number and aberrations (if any).

Disclaimer: This assay was conducted solely for the listed investigator/institution. The results of this assay are for research use only.

ThermoFisher SCIENTIFIC

KaryoStat™ Results: CUB12899-26

1. KaryoStat™ analysis of CUB12899-26 revealed the sample originated from a female individual
2. No chromosomal aberrations were found when comparing against the reference dataset (Figure 16)

Copy Number State

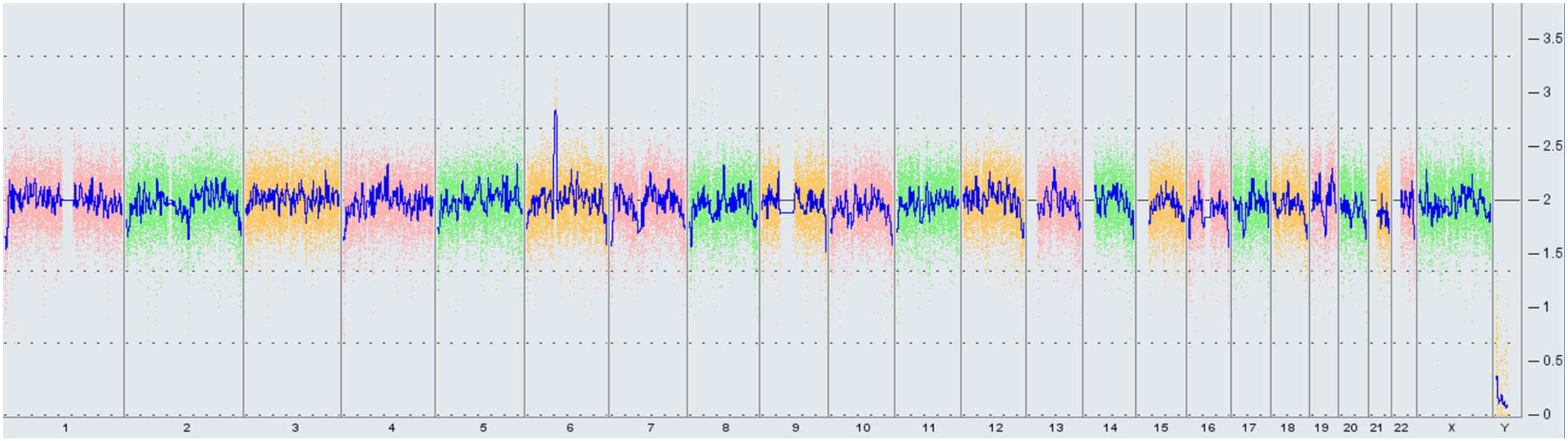

Figure 16: Whole genome view. The whole genome view displays all somatic and sex chromosomes in one frame with high level copy number. The smooth signal plot (right y-axis) is the smoothing of the log2 ratios which depict the signal intensities of probes on the microarray. A value of 2 represents a normal copy number state (CN = 2). A value of 3 represents chromosomal gain (CN = 3). A value of 1 represents a chromosomal loss (CN = 1). The pink, green and yellow colors indicate the raw signal for each individual chromosome probe, while the blue signal represents the normalized probe signal which is used to identify copy number and aberrations (if any).

Disclaimer: This assay was conducted solely for the listed investigator/institution. The results of this assay are for research use only.

ThermoFisher SCIENTIFIC

KaryoStat™ Results: CUB12899-27

1. KaryoStat™ analysis of CUB12899-27 revealed the sample originated from a female individual
2. No chromosomal aberrations were found when comparing against the reference dataset (Figure 17)

Copy Number State

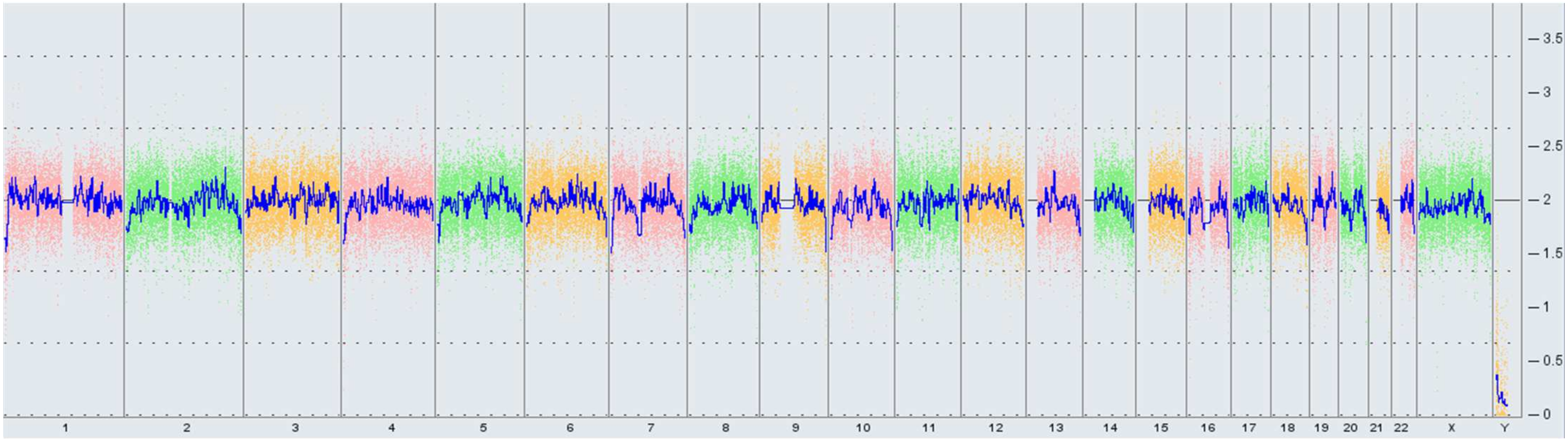

Figure 17: Whole genome view. The whole genome view displays all somatic and sex chromosomes in one frame with high level copy number. The smooth signal plot (right y-axis) is the smoothing of the log2 ratios which depict the signal intensities of probes on the microarray. A value of 2 represents a normal copy number state (CN = 2). A value of 3 represents chromosomal gain (CN = 3). A value of 1 represents a chromosomal loss (CN = 1). The pink, green and yellow colors indicate the raw signal for each individual chromosome probe, while the blue signal represents the normalized probe signal which is used to identify copy number and aberrations (if any).

Disclaimer: This assay was conducted solely for the listed investigator/institution. The results of this assay are for research use only.

ThermoFisher SCIENTIFIC

KaryoStat™ Results: CUB12899r-1

1. KaryoStat™ analysis of CUB12899r-1 revealed the sample originated from a male individual
2. Chromosomal aberrations were observed when comparing to the reference set (Table 2, Figure 1)
3. A supplemental document with detailed information on the aberration will be provided to the Client

Chromosome	Type	Cytoband Start	CN State	Size (kbp)
2	Gain	q37.3	3	2,546
10	Loss	p15.3	1	17,004
20	Loss	p13	1	25,696
20	Gain	p11.1	3	36,035
X	Gain	p22.33	3	154,679

Table 2: KaryoStat™ analysis. Chromosomal aberrations are indicated in the table shown. See supplemental data for more details.

Copy Number State

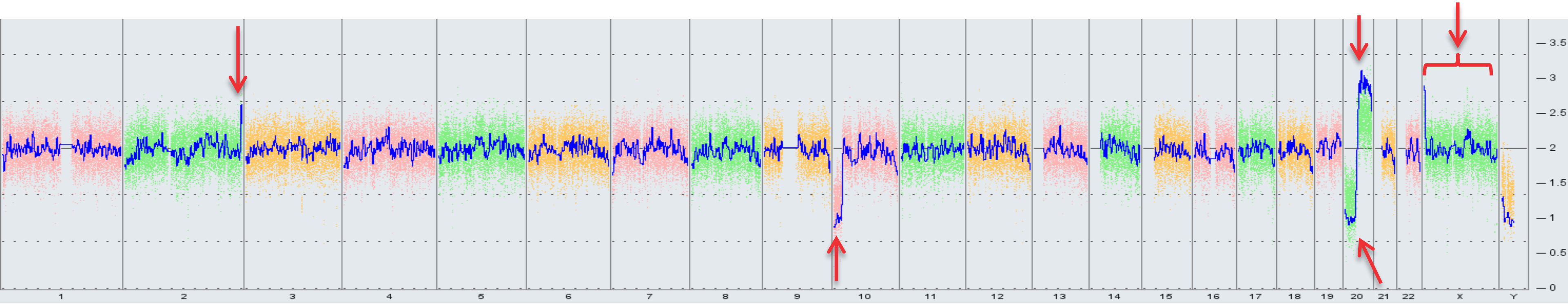

Figure 1: Whole genome view. The whole genome view displays all somatic and sex chromosomes in one frame with high level copy number. The smooth signal plot (right y-axis) is the smoothing of the log2 ratios which depict the signal intensities of probes on the microarray. A value of 2 represents a normal copy number state (CN = 2). A value of 3 represents chromosomal gain (CN = 3). A value of 1 represents a chromosomal loss (CN = 1). The pink, green and yellow colors indicate the raw signal for each individual chromosome probe, while the blue signal represents the normalized probe signal which is used to identify copy number and aberrations (if any). The whole genome view analysis revealed chromosomal aberrations indicated by the red arrow.

Disclaimer: This assay was conducted solely for the listed investigator/institution. The results of this assay are for research use only.

ThermoFisher SCIENTIFIC

KaryoStat™ Results: CUB12899r-4

1. KaryoStat™ analysis of CUB12899r-4 revealed the sample originated from a male individual
2. Chromosomal aberrations were observed when comparing to the reference set (Table 3, Figure 3)
3. A supplemental document with detailed information on the aberration will be provided to the Client

Chromosome	Type	Cytoband Start	CN State	Size (kbp)
5	Gain	p15.33	3	30,024

Table 3: KaryoStat™ analysis. Chromosomal aberrations are indicated in the table shown. See supplemental data for more details.

Copy Number State

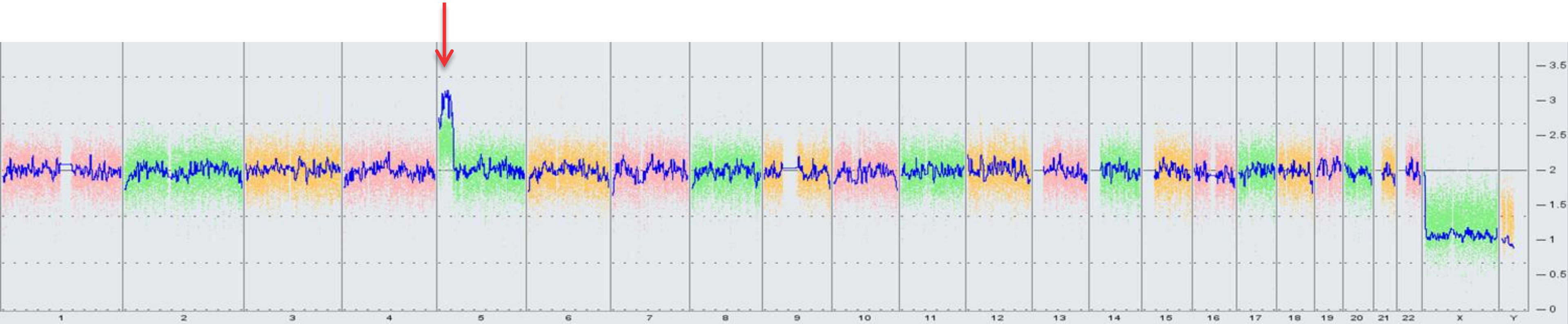

Figure 3: Whole genome view. The whole genome view displays all somatic and sex chromosomes in one frame with high level copy number. The smooth signal plot (right y-axis) is the smoothing of the log2 ratios which depict the signal intensities of probes on the microarray. A value of 2 represents a normal copy number state (CN = 2). A value of 3 represents chromosomal gain (CN = 3). A value of 1 represents a chromosomal loss (CN = 1). The pink, green and yellow colors indicate the raw signal for each individual chromosome probe, while the blue signal represents the normalized probe signal which is used to identify copy number and aberrations (if any). The whole genome view analysis revealed chromosomal aberrations indicated by the red arrow.

Disclaimer: This assay was conducted solely for the listed investigator/institution. The results of this assay are for research use only.

ThermoFisher SCIENTIFIC

KaryoStat™ Results: CUB12899-18

1. KaryoStat™ analysis of CUB12899-18 revealed the sample originated from a male individual
2. No chromosomal aberrations were found when comparing against the reference dataset (Figure 10)

Copy Number State

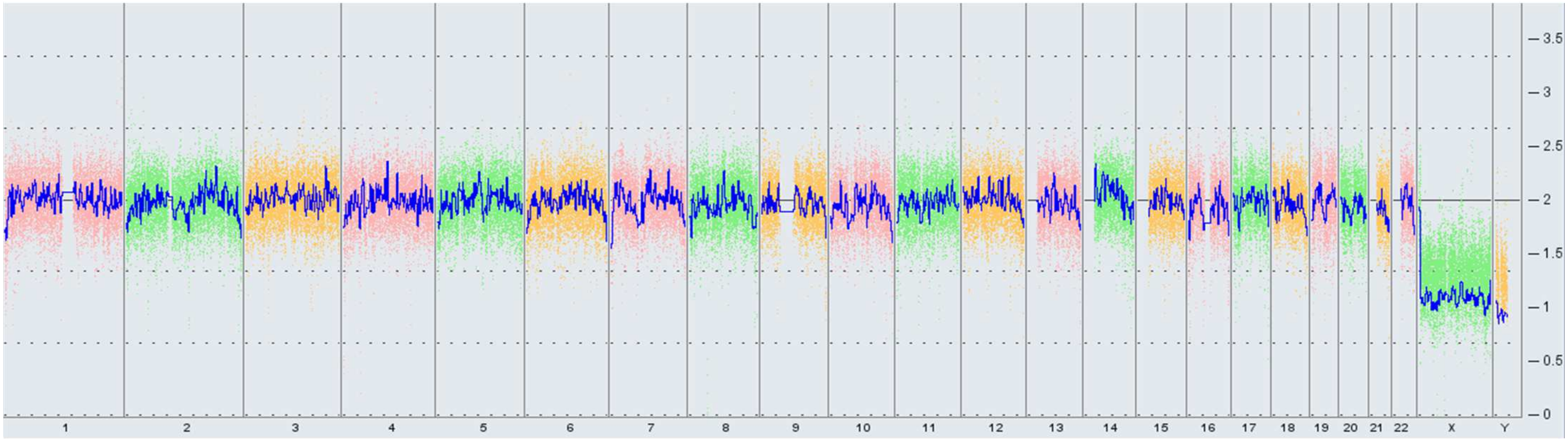

Figure 10: Whole genome view. The whole genome view displays all somatic and sex chromosomes in one frame with high level copy number. The smooth signal plot (right y-axis) is the smoothing of the log2 ratios which depict the signal intensities of probes on the microarray. A value of 2 represents a normal copy number state (CN = 2). A value of 3 represents chromosomal gain (CN = 3). A value of 1 represents a chromosomal loss (CN = 1). The pink, green and yellow colors indicate the raw signal for each individual chromosome probe, while the blue signal represents the normalized probe signal which is used to identify copy number and aberrations (if any).

Disclaimer: This assay was conducted solely for the listed investigator/institution. The results of this assay are for research use only.

KaryoStat™ Results: CUB12899-9

1. KaryoStat™ analysis of CUB12899-9 revealed the sample originated from a male individual
2. No chromosomal aberrations were found when comparing against the reference dataset (Figure 4)

Copy Number State

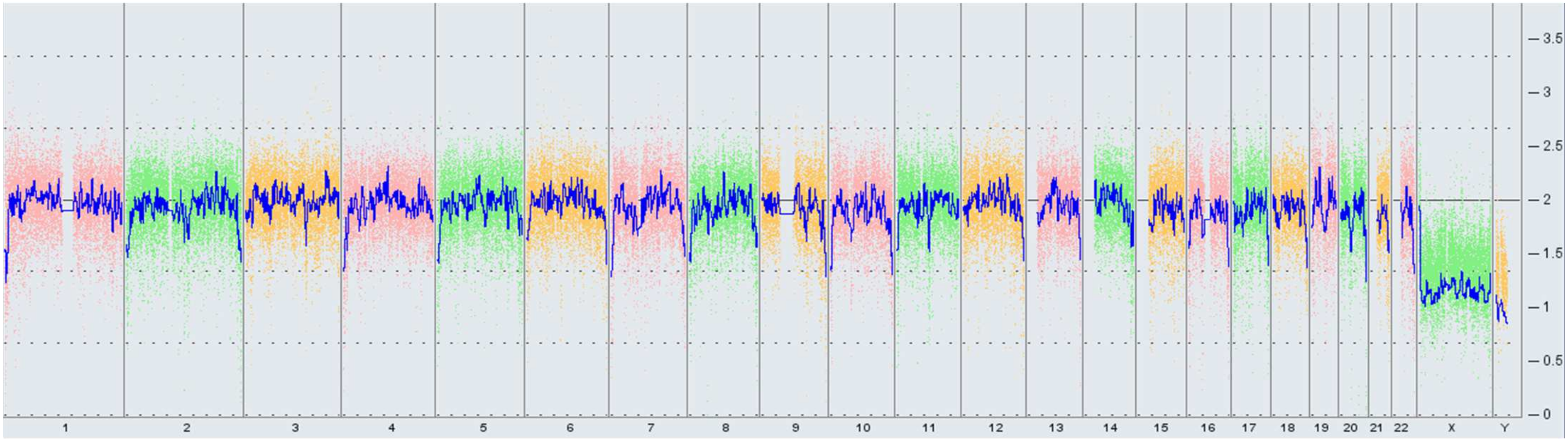

Figure 4: Whole genome view. The whole genome view displays all somatic and sex chromosomes in one frame with high level copy number. The smooth signal plot (right y-axis) is the smoothing of the log2 ratios which depict the signal intensities of probes on the microarray. A value of 2 represents a normal copy number state (CN = 2). A value of 3 represents chromosomal gain (CN = 3). A value of 1 represents a chromosomal loss (CN = 1). The pink, green and yellow colors indicate the raw signal for each individual chromosome probe, while the blue signal represents the normalized probe signal which is used to identify copy number and aberrations (if any).

Disclaimer: This assay was conducted solely for the listed investigator/institution. The results of this assay are for research use only.

ThermoFisher SCIENTIFIC

KaryoStat™ Results: CUB12899-10

1. KaryoStat™ analysis of CUB12899-10 revealed the sample originated from a male individual
2. No chromosomal aberrations were found when comparing against the reference dataset (Figure 5)

Copy Number State

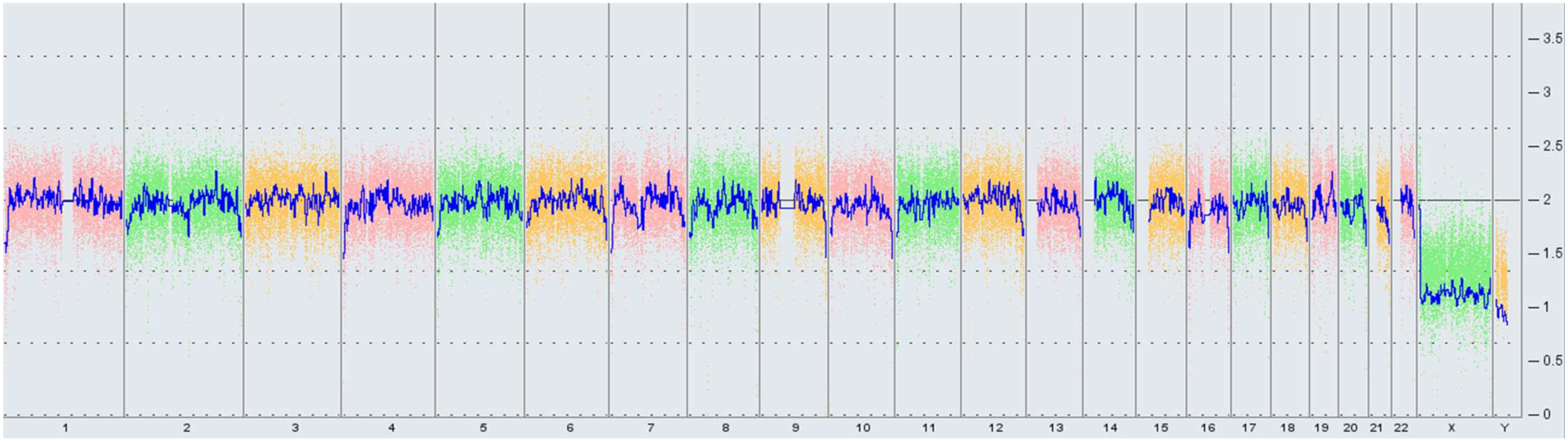

Figure 5: Whole genome view. The whole genome view displays all somatic and sex chromosomes in one frame with high level copy number. The smooth signal plot (right y-axis) is the smoothing of the log2 ratios which depict the signal intensities of probes on the microarray. A value of 2 represents a normal copy number state (CN = 2). A value of 3 represents chromosomal gain (CN = 3). A value of 1 represents a chromosomal loss (CN = 1). The pink, green and yellow colors indicate the raw signal for each individual chromosome probe, while the blue signal represents the normalized probe signal which is used to identify copy number and aberrations (if any).

Disclaimer: This assay was conducted solely for the listed investigator/institution. The results of this assay are for research use only.

ThermoFisher SCIENTIFIC

KaryoStat™ Results: CUB12899-11

1. KaryoStat™ analysis of CUB12899-11 revealed the sample originated from a male individual
2. No chromosomal aberrations were found when comparing against the reference dataset (Figure 6)

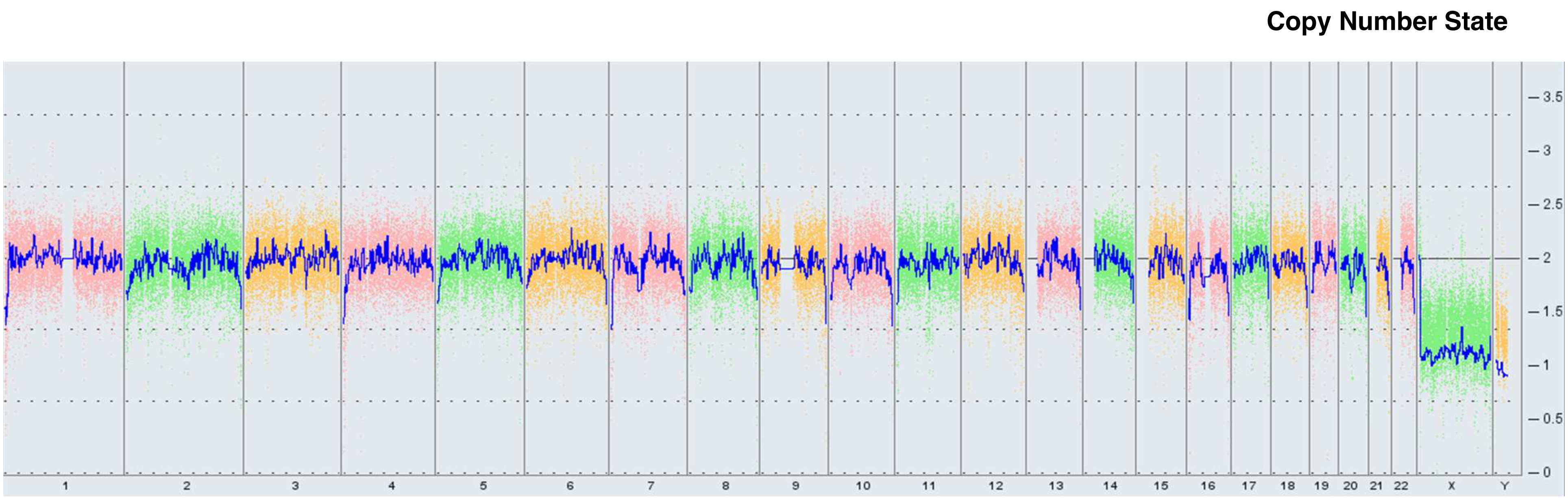

Figure 6: Whole genome view. The whole genome view displays all somatic and sex chromosomes in one frame with high level copy number. The smooth signal plot (right y-axis) is the smoothing of the log2 ratios which depict the signal intensities of probes on the microarray. A value of 2 represents a normal copy number state (CN = 2). A value of 3 represents chromosomal gain (CN = 3). A value of 1 represents a chromosomal loss (CN = 1). The pink, green and yellow colors indicate the raw signal for each individual chromosome probe, while the blue signal represents the normalized probe signal which is used to identify copy number and aberrations (if any).

Disclaimer: This assay was conducted solely for the listed investigator/institution. The results of this assay are for research use only.

KaryoStat™ Results: CUB12899-8

1. KaryoStat™ analysis of CUB12899-8 revealed the sample originated from a male individual
2. Chromosomal aberrations were observed when comparing to the reference set (Table 3, Figure 3)
3. A supplemental document with detailed information on the aberration will be provided to the Client

Chromosome	Type	Cytoband Start	CN State	Size (kbp)
2	Gain	q33.2	3	2,618

Table 3: KaryoStat™ analysis. Chromosomal aberrations are indicated in the table shown**.** See supplemental data for more details.

Copy Number State

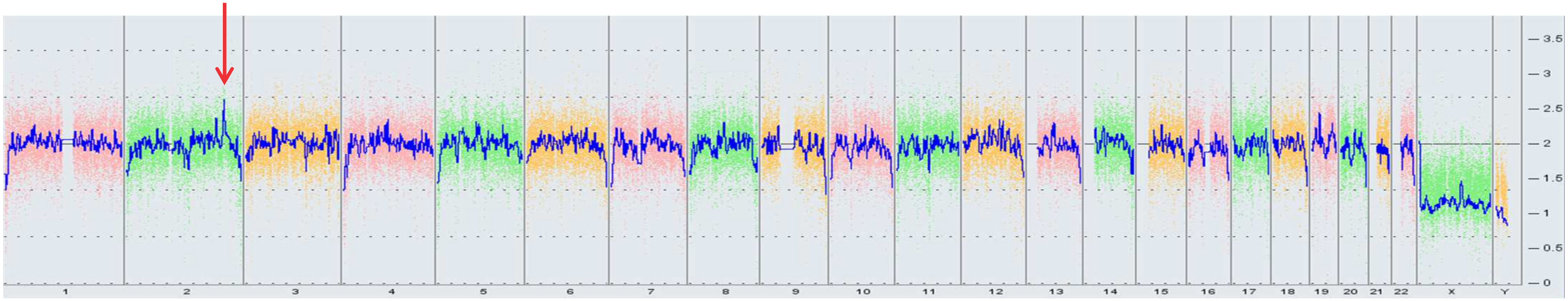

Figure 3: Whole genome view. The whole genome view displays all somatic and sex chromosomes in one frame with high level copy number. The smooth signal plot (right y-axis) is the smoothing of the log2 ratios which depict the signal intensities of probes on the microarray. A value of 2 represents a normal copy number state (CN = 2). A value of 3 represents chromosomal gain (CN = 3). A value of 1 represents a chromosomal loss (CN = 1). The pink, green and yellow colors indicate the raw signal for each individual chromosome probe, while the blue signal represents the normalized probe signal which is used to identify copy number and aberrations (if any). The whole genome view analysis revealed chromosomal aberrations indicated by the red arrow.

Disclaimer: This assay was conducted solely for the listed investigator/institution. The results of this assay are for research use only.

ThermoFisher SCIENTIFIC

KaryoStat™ Results: CUB12899-21

1. KaryoStat™ analysis of CUB12899-21 revealed the sample originated from a female individual
2. No chromosomal aberrations were found when comparing against the reference dataset (Figure 22)

Copy Number State

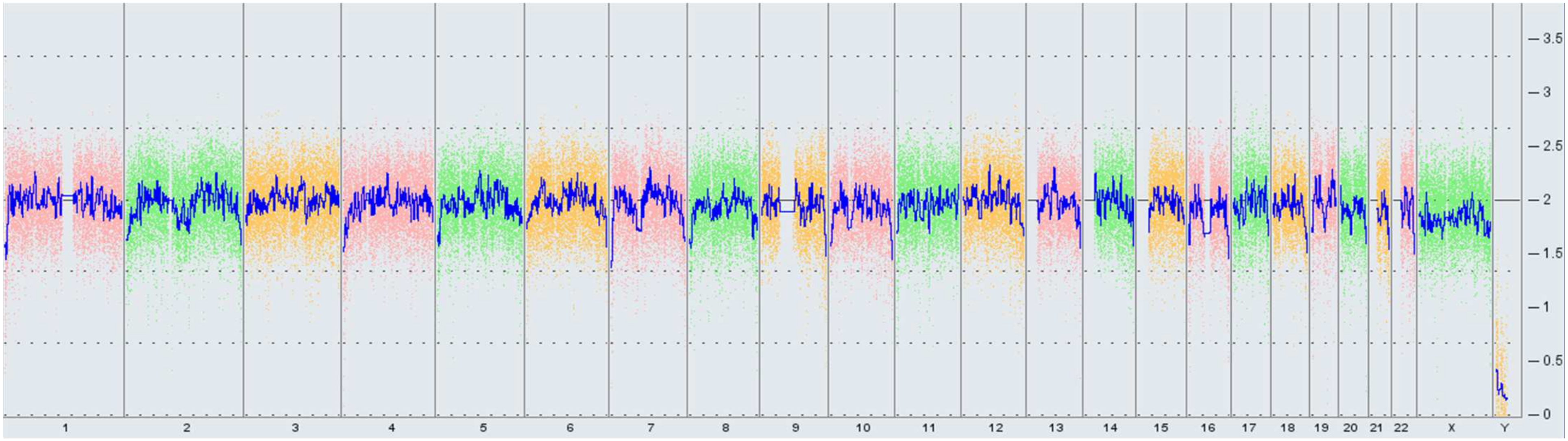

Figure 22: Whole genome view. The whole genome view displays all somatic and sex chromosomes in one frame with high level copy number. The smooth signal plot (right y-axis) is the smoothing of the log2 ratios which depict the signal intensities of probes on the microarray. A value of 2 represents a normal copy number state (CN = 2). A value of 3 represents chromosomal gain (CN = 3). A value of 1 represents a chromosomal loss (CN = 1). The pink, green and yellow colors indicate the raw signal for each individual chromosome probe, while the blue signal represents the normalized probe signal which is used to identify copy number and aberrations (if any).

Disclaimer: This assay was conducted solely for the listed investigator/institution. The results of this assay are for research use only.

Thermo Fisher SCIENTIFIC

KaryoStat™ Results: CUB12899-22

1. KaryoStat™ analysis of CUB12899-22 revealed the sample originated from a female individual
2. No chromosomal aberrations were found when comparing against the reference dataset (Figure 23)

Copy Number State

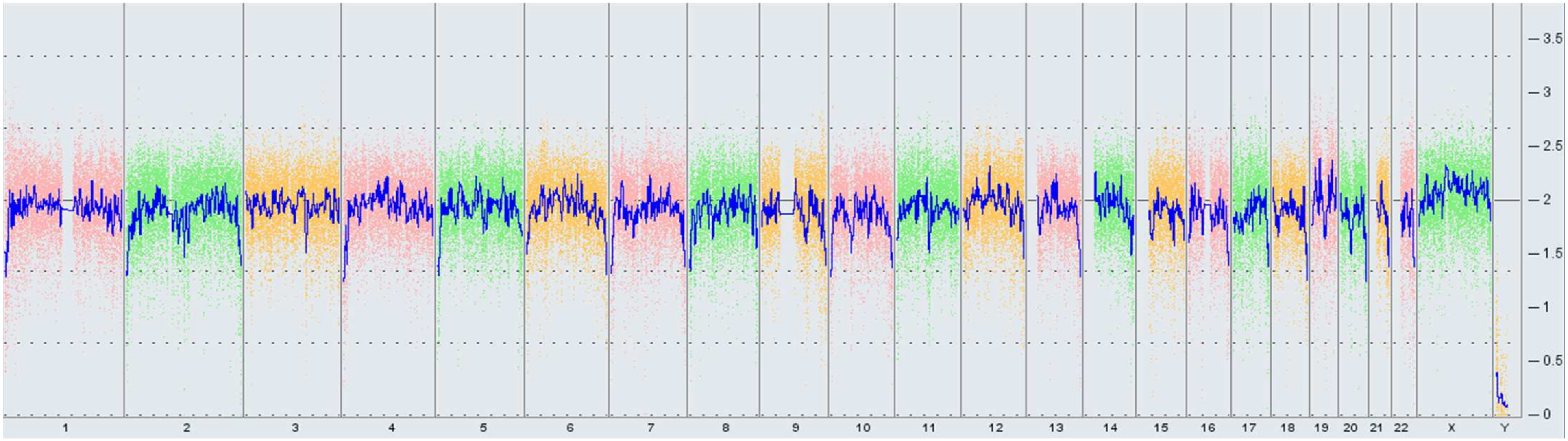

Figure 23: Whole genome view. The whole genome view displays all somatic and sex chromosomes in one frame with high level copy number. The smooth signal plot (right y-axis) is the smoothing of the log2 ratios which depict the signal intensities of probes on the microarray. A value of 2 represents a normal copy number state (CN = 2). A value of 3 represents chromosomal gain (CN = 3). A value of 1 represents a chromosomal loss (CN = 1). The pink, green and yellow colors indicate the raw signal for each individual chromosome probe, while the blue signal represents the normalized probe signal which is used to identify copy number and aberrations (if any).

Disclaimer: This assay was conducted solely for the listed investigator/institution. The results of this assay are for research use only.
