# Supplementary material for: Transcriptome analysis and functional characterization of cerebral organoids in bipolar disorder
Source: Genome Med. 2020 Apr 19;12:34. doi: 10.1186/s13073-020-00733-6 (PMC7168850; doi:10.1186/s13073-020-00733-6)

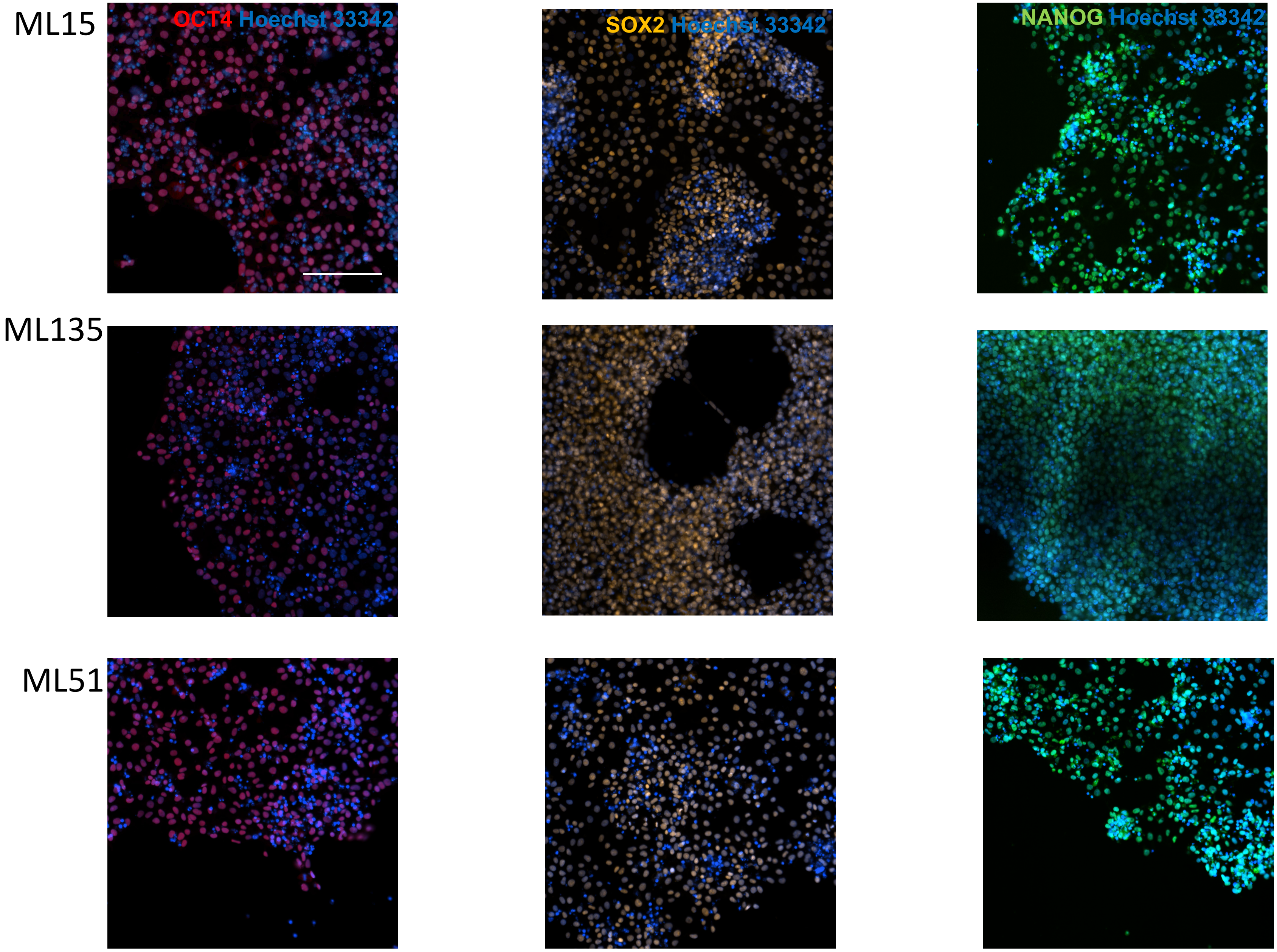
ML15
ML135
ML51
OCT4 Hoechst 33342
SOX2 Hoechst 33342
NANOG Hoechst 33342

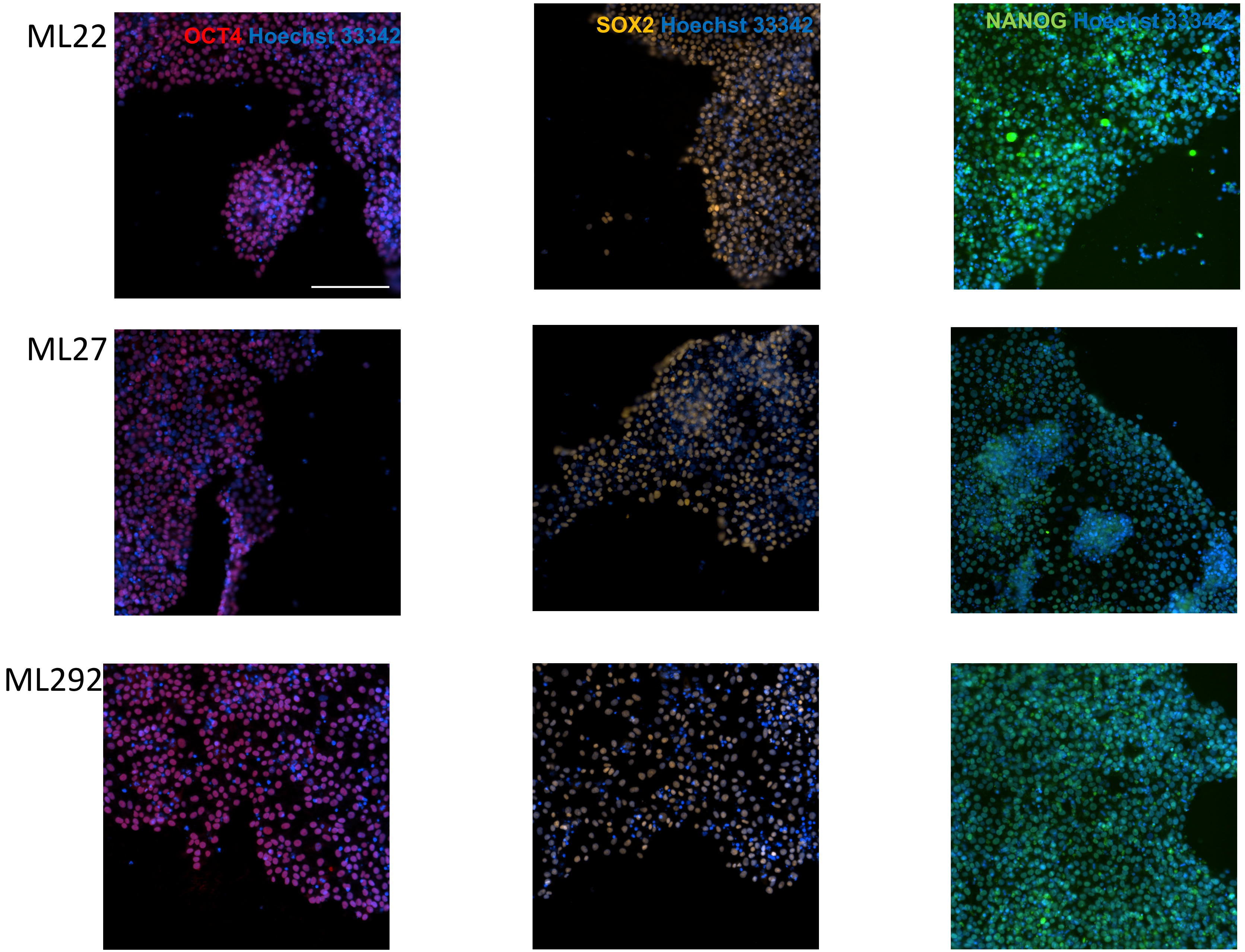
ML22
OCT4 Hoechst 33342
SOX2 Hoechst 33342
NANOG Hoechst 33342
ML27
ML292

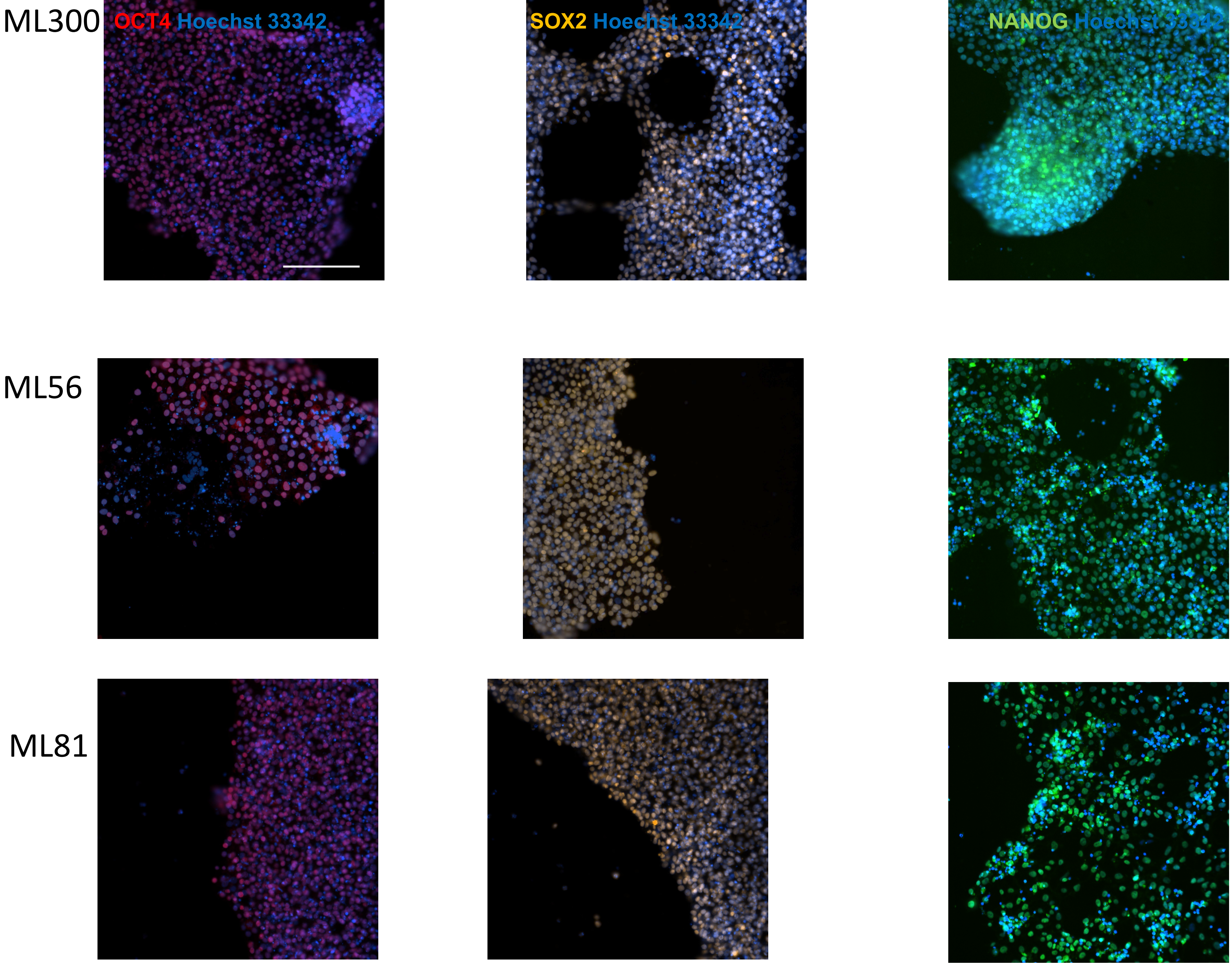
ML300
OCT4 Hoechst 33342
SOX2 Hoechst 33342
NANOG Hoechst 33342
ML56
ML81

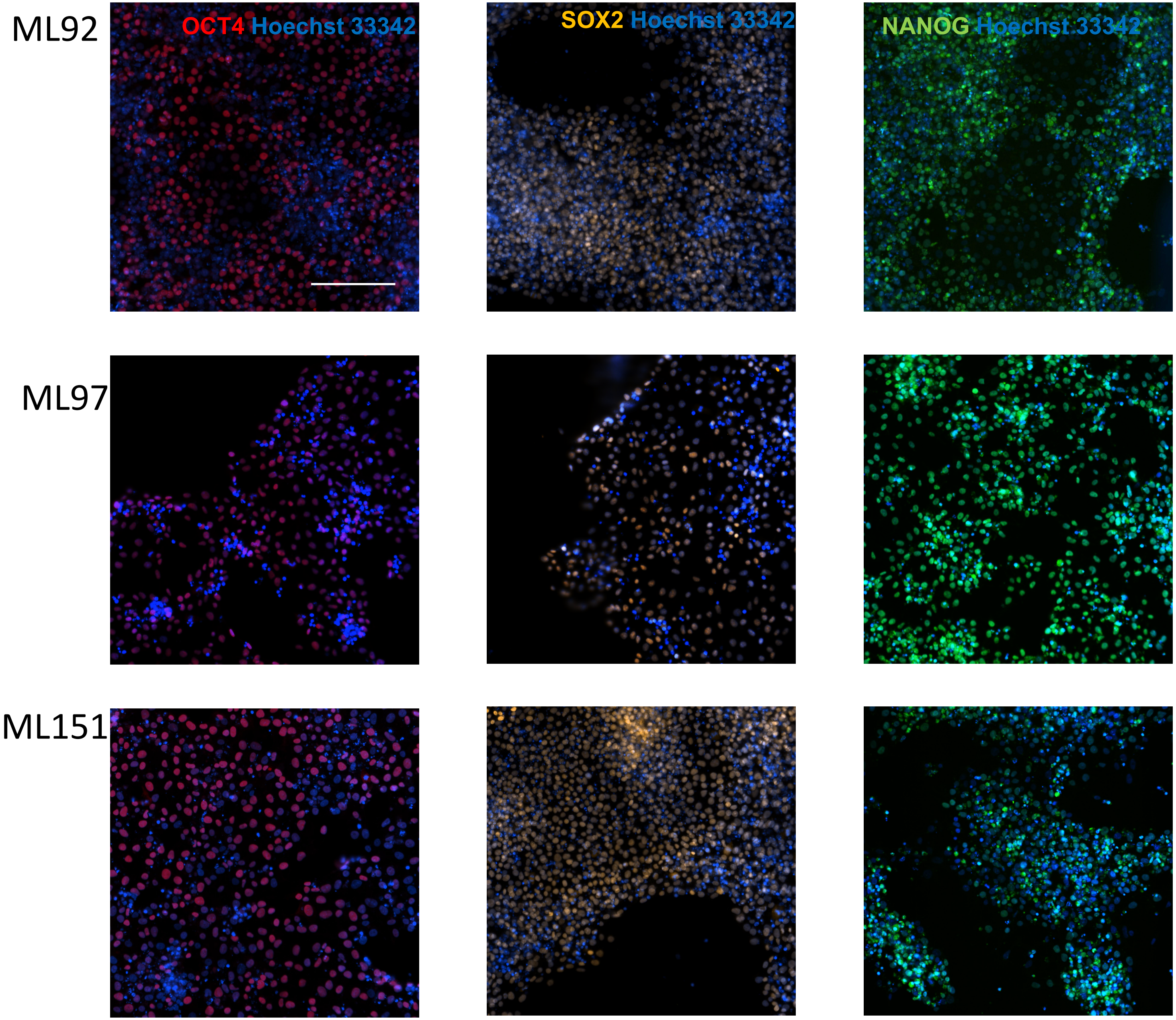

ML92
ML97
ML151
OCT4 Hoechst 33342
SOX2 Hoechst 33342
NANOG Hoechst 33342

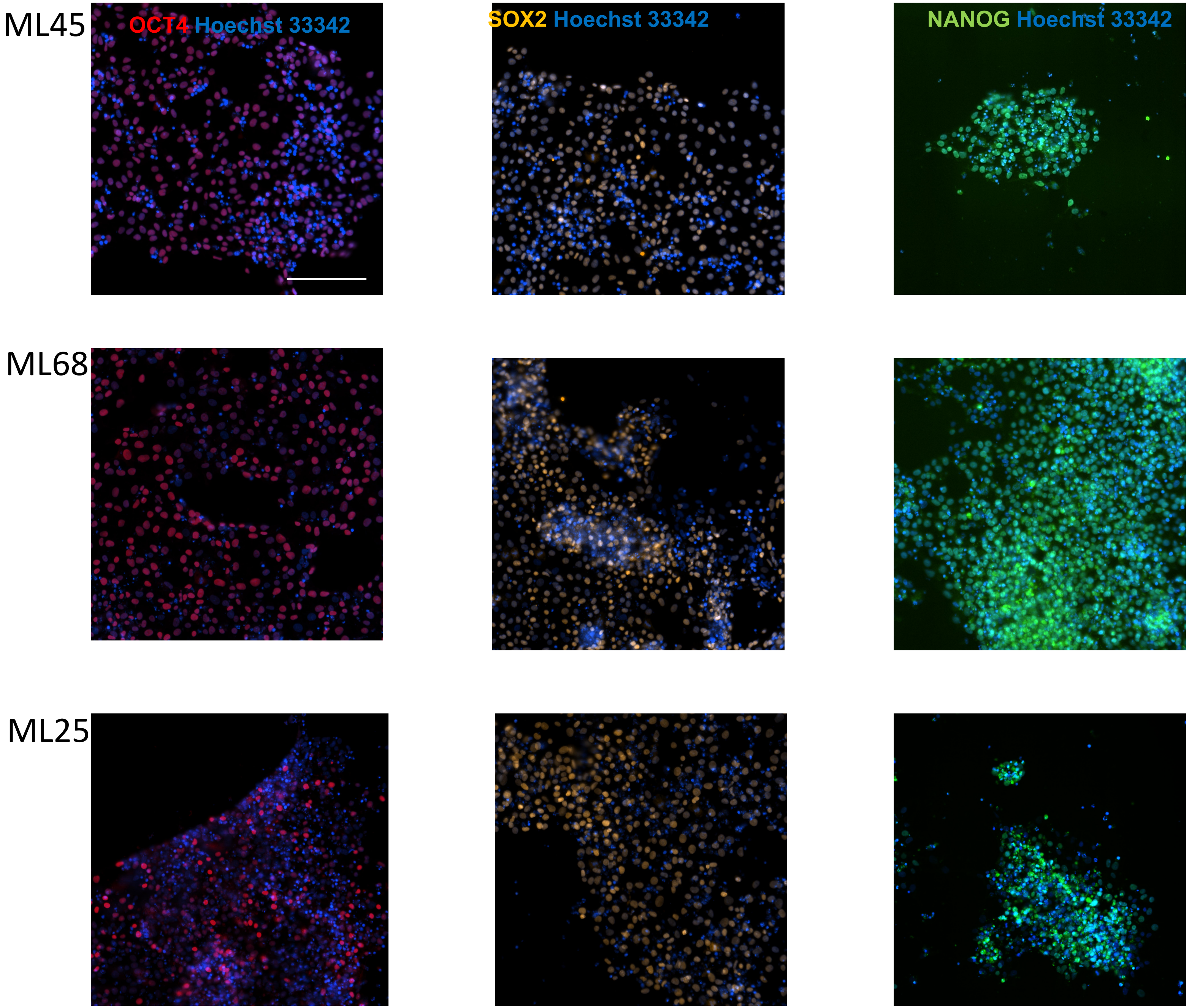
ML45
OCT4 Hoechst 33342
SOX2 Hoechst 33342
NANOG Hoechst 33342
ML68
ML25

ML136

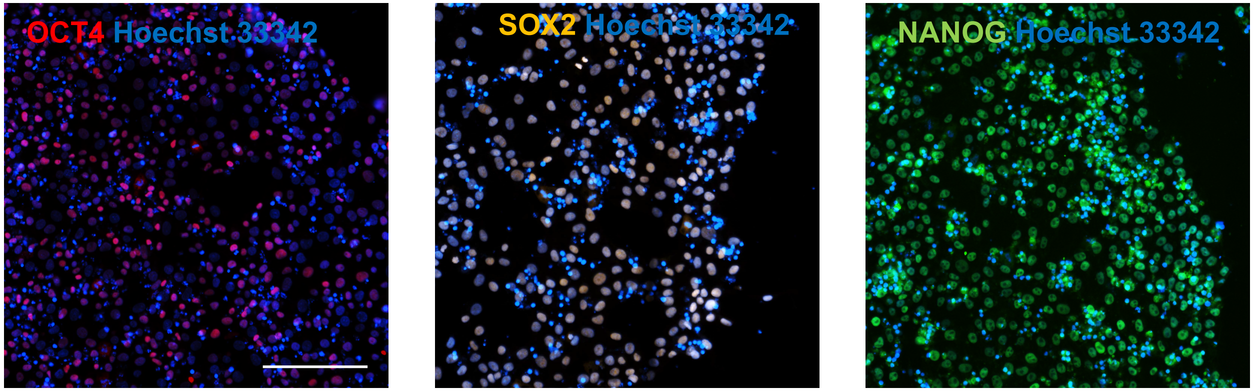

Supplement: Supplementary file 3 — Additional file 3. Marker analysis for iPSC lines. [file 13073_2020_733_MOESM3_ESM.pdf]
